# Supplementary material for: Calpeptin is a potent cathepsin inhibitor and drug candidate for SARS-CoV-2 infections
Source: Commun Biol. 2023 Oct 18;6:1058. doi: 10.1038/s42003-023-05317-9 (PMC10584882; doi:10.1038/s42003-023-05317-9)
Supplement: Supplementary file 2 — Supplemental text accepted [file 42003_2023_5317_MOESM2_ESM.pdf]

Supplementary information for

Calpeptin is a potent cathepsin inhibitor and drug candidate for SARS-CoV-2 infections

Patrick YA Reinke[1]†, Edmarcia Elisa de Souza[2]†, Sebastian Günther[1]†, Sven Falke[1], Julia Lieske[1], Wiebke Ewert[1], Jure Loboda[3,4], Alexander Herrmann[5], Aida Rahmani Mashhour[1], Katarina Karničar[3,6], Aleksandra Usenik[3,6], Nataša Lindič[3], Andreja Sekirnik[3], Viviane Fongaro Botosso[7], Gláucia Maria Machado Santelli[8], Josana Kapronezai[7], Marcelo Valdemir de Araújo[7,9], Taiana Tainá Silva-Pereira[9,10], Antônio Francisco de Souza Filho[9], Mariana Silva Tavares[9], Lizdany Flórez-Álvarez[2], Danielle Bruna Leal de Oliveira[9], Edison Luiz Durigon[9], Paula Roberta Giaretta[11,] Marcos Bryan Heinemann[10], Maurice Hauser[12], Brandon Seychell[13], Hendrik Böhler[13], Wioletta Rut[14], Marcin Drag[14], Tobias Beck[13,15], Russell Cox[12], Henry N Chapman[1,15,16], Christian Betzel[15,17], Wolfgang Brehm[1], Winfried Hinrichs[18], Gregor Ebert[5,19], Sharissa L Latham[20, 21], Ana Marcia de Sá Guimarães[9], Dusan Turk[3,6]*, Carsten Wrenger[2]*, Alke Meents[1]*

*Corresponding author. Email: dusan.turk@ijs.si, cwrenger@icb.usp.br, alke.meents@desy.de

This PDF file includes:

Supplementary Text

Figs. S1 to S12, Tables S1

Supplementary Text

Supplementary Note 1: Detailed description of the X-ray crystal structures of M^{pro} inhibitor complexes

The structures of Calpeptin and GC-376 bound to M^{pro} were previously described[1–4]. Calpeptin binds covalently to Cys^{145} of M^{pro} (PDB: 7AKU)[1] with its aldehyde group, forming a thiohemiacetal with an interatomic S-O distance of 1.8 Å (Figure S2F)[5,6]. This structure was solved in a monoclinic space group, where Calpeptin occupies the S1 to S3 pockets. Overall, the

peptidomimetic backbone of Calpeptin forms hydrogen bonds corresponding to the native peptide substrate[7]. The backbone carbonyl of His^{164} forms a hydrogen bond to the peptidomimetic Calpeptin amide-nitrogen of the norleucine residue-analog (Figure S12). The terminal ester carbonyl forms a hydrogen bond with the backbone nitrogen of Glu^{166}. The norleucine side chain exhibits van der Waals contacts with the backbone of Phe^{140}, Leu^{141}, and Asn^{142}. The terminal Cbz-group is oriented to the S3 pocket between the side chains of Glu^{166} and Gln^{189} and exhibits hydrophobic interactions with norleucine (Figure S2F). The structure of GC-376 aldehyde bound to M^{pro} solved in a monoclinic space group (PDB: 7QKA) resembles this conformation. Substitution of norleucine by 2-oxopyrrolidine leads to additional hydrogen bonds to His^{163} and Glu^{166}(Figure S2H). In addition to the monoclinic form with one monomer per asymmetric unit, M^{pro} also crystallizes in an orthorhombic space group containing two monomers per asymmetric unit[8] and the two monomers within the dimer exhibit structural variations. The structure of Calpeptin derived from S-Calpeptin bound to M^{pro} was solved in an orthorhombic space group (PDB 7Z3U) and shows more flexibility with respect to the conformation of the Cbz- group (Figure S2D). This group is oriented in the S4 pocket in one monomer, while it exhibits an alternative conformation oriented to the S3 or S4 pocket in the other monomer, a similar flexibility as observed for GC-376 analogs[9]. This is linked to an alternative orientation of the loop containing Gln^{189}, which is shifted by about 2 Å, further opening this pocket. In addition to the previously described interactions of Calpeptin, the Cbz-group interactions via hydrophobic interactions with the backbone of Thr^{190} and Gln^{192} are evident in the S4 pocket. In the- structure co-crystallized with S-Calpeptin, the monomers exhibit a NOS-bridge, which is an oxygen-mediated link between Cys^{22} and Lys^{61} (molecule B) and a SONOS-bridge, which is a three-way covalent bond between Cys^{22}, Cys^{44} and Lys^{61} (molecule A). These covalent links in M^{pro} were previously observed[10].

Supplementary Note 2: Detailed description of the X-ray crystal structures of CatK/V Calpeptin complex

The binding mode of Calpeptin to CatK (PDB: 8C3D) and CatV (PDB: 7QGW) is similar to that of CatL, which can be explained by the high conservation of the active site cleft (Figure S12). Small differences occur in the S3-pocket in the vicinity of the terminal Cbz-group of Calpeptin. While in CatL Leu^{69} and Tyr^{72} form a hydrophobic pocket in which the phenyl ring is located, this hydrophobic pocket is less voluminous in CatK because it is constrained by Tyr^{67} (in CatL Leu^{69}), which is oriented toward the Cbz-group, resulting in a restriction of the pocket that forces the phenyl ring towards Asp^{61} (in CatL Glu^{63}). CatV Phe^{69} and Arg^{72} (in CatL Leu^{69} and Tyr^{72}) are directed towards this pocket, leading to a restriction of the pocket and forcing the phenyl ring into a slightly different orientation towards Gln^{63} (in CatL Glu^{63}).

Supplementary Note 3: Analysis of TMPRSS2 expression by Western blot

TMPRSS2 is synthesized as a single-chain zymogen and cleaves SARS-CoV-2 S protein extracellularly. To determinate the expression status of endogenous TMPRSS2 during growth phase we treated LC-HK2 and VERO-CCL81 cells with trypsin to remove surface proteins. TMPRSS2 was barely detectable as a single band in VERO-CCL81 and detectable in LC-HK2 as ~ 54-kDa band, representing the zymogen (Figure S4). We detected two additional prominent bands with about ~64 kDa and ~110 kDa in human LC-HK2 probably generated by glycosylation events under reducing conditions in human TMPRSS2, as previously reported (Figure S4)[11].

Supplementary Note 4: Calpeptin cell experiments

To test whether the viral particles are entrapped in endosomes after S-Calpeptin treatment, the distribution of S-protein containing vesicle size in infected LC-HK2 cells was analyzed by

fluorescence microscopy. This analysis did not reveal significant differences between S-Calpeptin treated cells and the control group (Figure S5).

Supplementary Note 5: Calpeptin toxicity experiments in hamsters

At the time of this study, we did not find any study that used Calpeptin in hamsters. Therefore, prior to the antiviral experiment, we performed a small study to investigate if different doses of Calpeptin could lead to acute toxicity in hamsters. Animals were subjected to daily subcutaneous as described in the methods section. The route of administration and doses were chosen based on literature reports of the use of Calpeptin in mouse studies[12–18]. Clinical signs and weight were followed until day 6, when the animals were euthanized, necropsied, and organs were collected for histopathology analysis. Treated animals did not display acute toxicity-related clinical signs or weight loss during the experiment (Figure S8A and B) nor any noteworthy histopathological lesion in brain, liver, pancreas, gastro-intestinal tract, kidney, lungs, spleen, or trachea. However, a complete blood cell count performed on day 6 showed marked leukopenia caused by lymphopenia and neutropenia in animals treated with 2 mg/kg and 3 mg/kg of S-Calpeptin compared to those that received only 1 mg/kg of S-Calpeptin or 1:100 DMSO (Figure S8C-F). For this reason, we opted to test the dose of 1 mg/kg of S-Calpeptin in the anti-viral animal experiment (Figure S8A and B).

Supplementary Note 6: Histopathological lesions in hamsters infected with SARS-CoV-2

Corroborating previous studies[19–22], typical histopathological lesions were seen in hamsters inoculated with SARS-CoV-2 (G1 and G2). Findings in the lungs included bronchointerstitial pneumonia, suppurative bronchitis, endothelialitis, necrosis of epithelial cells, and hyperplasia of bronchial and alveolar type II epithelial cells (Figure S6). Occasional atypical syncytial cells were seen. Areas of interstitial pulmonary fibrosis and/or inflammation were occasionally detected on

both infected groups. On day 7 p.i., there were higher tissue repair scores in infected animals (Figure S6F), coinciding with a lower viral load in respiratory tissues and beginning of the recovery phase (Figure 5). Lesions in the trachea of infected hamsters consisted of loss of cilia, degeneration and/or necrosis of epithelial cells and inflammatory infiltrate in the lumen and/or in the lamina propria. Histopathology analysis showed no statistically significant differences in lesion scores between SARS-CoV-2 infected animals that were treated with S-Calpeptin and to those that were not (Figure S7). Lung vascular lesions are a hallmark of COVID-19 in hamsters[21]. Although S-Calpeptin-treated animals showed lower scores for vascular damage compared to the untreated group on days 5 and 7 p.i., these results were also not statistically significant (Figure S7). As expected, the lungs and trachea of hamsters from control groups (G3 and G4) were unremarkable.

References

1. Günther, S. *et al.* X-ray screening identifies active site and allosteric inhibitors of SARS-CoV-2 main protease. *Science* **372**, 642–646 (2021).
2. Vuong, W. *et al.* Feline coronavirus drug inhibits the main protease of SARS-CoV-2 and blocks virus replication. *Nat Commun* **11**, 4282 (2020).
3. Fu, L. *et al.* Both Boceprevir and GC376 efficaciously inhibit SARS-CoV-2 by targeting its main protease. *Nat Commun* **11**, 4417 (2020).
4. Ma, C. *et al.* Boceprevir, GC-376, and calpain inhibitors II, XII inhibit SARS-CoV-2 viral replication by targeting the viral main protease. *Cell Res* **30**, 678–692 (2020).
5. Ramos-Guzmán, C. A., Ruiz-Pernía, J. J. & Tuñón, I. Multiscale Simulations of SARS-CoV-2 3CL Protease Inhibition with Aldehyde Derivatives. Role of Protein and Inhibitor Conformational Changes in the Reaction Mechanism. *ACS Catal.* **11**, 4157–4168 (2021).
6. Kuchitsu, K., Fukuyama, T., Tamaru, Y., Yoshida, Z. & Tabushi, I. Carbon-sulfur bond distances and sulfur valence angles in 5-thiabicyclo[2.1.1]hexane and 7-thiabicyclo[2.2.1]heptane as determined by gas electron diffraction. *J. Am. Chem. Soc.* **93**, 2799–2800 (1971).
7. Lee, J. *et al.* Crystallographic structure of wild-type SARS-CoV-2 main protease acyl-enzyme intermediate with physiological C-terminal autoprocessing site. *Nat Commun* **11**, 5877 (2020).
8. Zhang, L. *et al.* Crystal structure of SARS-CoV-2 main protease provides a basis for design of improved α-ketoamide inhibitors. *Science* **368**, 409–412 (2020).
9. Sacco, M. D. *et al.* Structure and inhibition of the SARS-CoV-2 main protease reveal strategy for developing dual inhibitors against Mpro and cathepsin L. *Sci Adv* **6**, eabe0751 (2020).
10. Rabe von Pappenheim, F. *et al.* Widespread occurrence of covalent lysine–cysteine redox switches in proteins. *Nat Chem Biol* 1–8 (2022) doi:10.1038/s41589-021-00966-5.
11. Zhang, Y. *et al.* Transmembrane serine protease TMPRSS2 implicated in SARS-CoV-2 infection is autoactivated intracellularly and requires N-glycosylation for regulation. *J Biol Chem* **298**, 102643 (2022).
12. Bandaru, S. *et al.* Targeting Filamin A Reduces Macrophage Activity and Atherosclerosis. *Circulation* **140**, 67–79 (2019).
13. Song, L.-J. *et al.* Inhibition of angiotensin II and calpain attenuates pleural fibrosis. *Pulmonary Pharmacology & Therapeutics* **48**, 46–52 (2018).

14. Tabata, C., Tabata, R. & Nakano, T. The calpain inhibitor calpeptin prevents bleomycin-induced pulmonary fibrosis in mice. *Clinical and Experimental Immunology* **162**, 560–567 (2010).

15. Yoshida, M. *et al.* Calpain inhibitor calpeptin suppresses pancreatic cancer by disrupting cancer-stromal interactions in a mouse xenograft model. *Cancer Sci* **107**, 1443–1452 (2016).

16. Mani, S. K. *et al.* Calpain inhibition preserves myocardial structure and function following myocardial infarction. *American Journal of Physiology-Heart and Circulatory Physiology* **297**, H1744–H1751 (2009).

17. Samantaray, S. *et al.* Inhibition of Calpain Activation Protects MPTP-Induced Nigral and Spinal Cord Neurodegeneration, Reduces Inflammation, and Improves Gait Dynamics in Mice. *Mol Neurobiol* **52**, 1054–1066 (2015).

18. Zuo, J. *et al.* Calpeptin attenuates cigarette smoke-induced pulmonary inflammation via suppressing calpain/IκBα signaling in mice and BEAS-2B cells. *Pathol Res Pract* **214**, 1199–1209 (2018).

19. Imai, M. *et al.* Syrian hamsters as a small animal model for SARS-CoV-2 infection and countermeasure development. *Proc Natl Acad Sci U S A* **117**, 16587–16595 (2020).

20. Chan, J. F.-W. *et al.* Simulation of the Clinical and Pathological Manifestations of Coronavirus Disease 2019 (COVID-19) in a Golden Syrian Hamster Model: Implications for Disease Pathogenesis and Transmissibility. *Clin Infect Dis* **71**, 2428–2446 (2020).

21. Gruber, A. D. *et al.* Standardization of Reporting Criteria for Lung Pathology in SARS-CoV-2–infected Hamsters: What Matters? *Am J Respir Cell Mol Biol* **63**, 856–859 (2020).

22. Sia, S. F. *et al.* Pathogenesis and transmission of SARS-CoV-2 in golden hamsters. *Nature* **583**, 834–838 (2020).

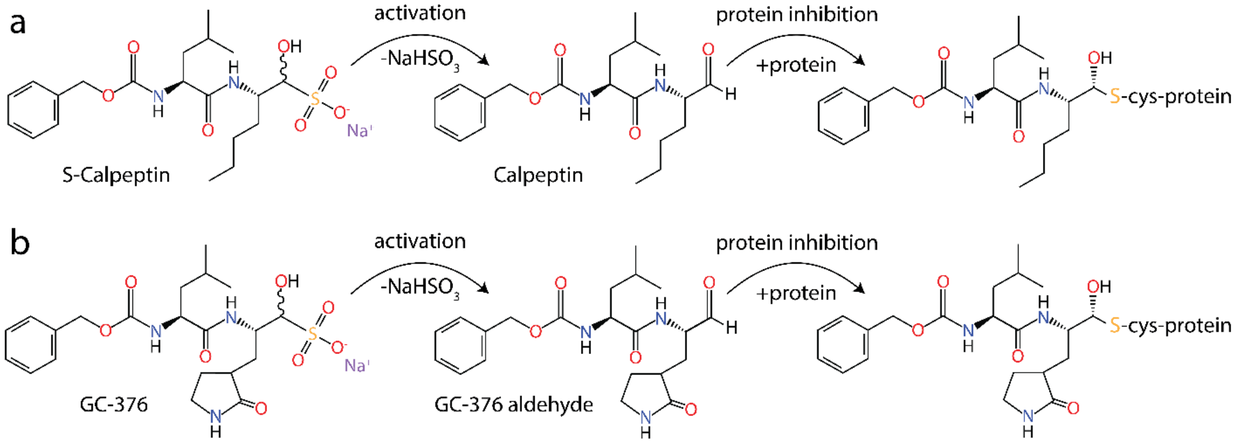

Figure S1.

Activation of the prodrugs S-Calpeptin and GC-376. (a) S-Calpeptin and (b) GC-376 contain a sulfonated aldehyde, which under physiological conditions slowly converts into hydrogensulfite and the aldehyde, thereby unmasking the warhead. The aldehyde warhead binds covalently to catalytic cysteines of cathepsins and M^{pro}.

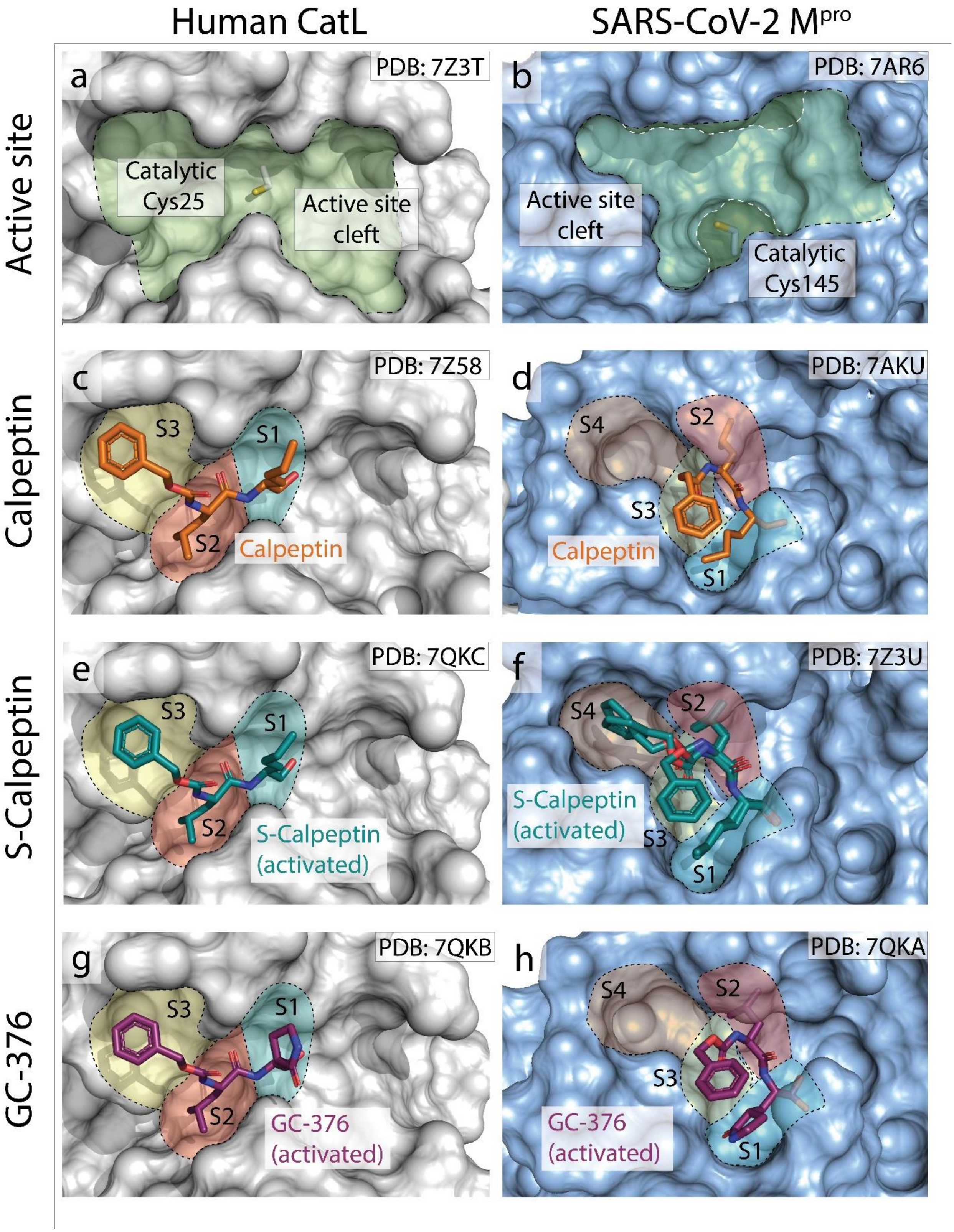

Human CatL
SARS-CoV-2 Mpro
Active site
Calpeptin
S-Calpeptin
GC-376
a
PDB: 7Z3T
Catalytic Cys25
Active site cleft
b
PDB: 7AR6
Active site cleft
Catalytic Cys145
c
PDB: 7Z58
S3
S1
S2
Calpeptin
d
PDB: 7AKU
S4
S2
S3
Calpeptin
S1
e
PDB: 7QKC
S3
S1
S2
S-Calpeptin (activated)
f
PDB: 7Z3U
S4
S2
S-Calpeptin (activated)
S3
S1
g
PDB: 7QKB
S3
S1
S2
GC-376 (activated)
h
PDB: 7QKA
S4
S2
S3
GC-376 (activated)
S1

168

169

Figure S2.

Comparison of inhibitor binding to the active sites of CatL and M^{pro}. Closeup view of the active site (green) of CatL (a, grey surface) and M^{pro} (b, blue surface). M^{pro} residues Ser^{46} and Asn^{142} are shown as a transparent area (white dashes, dark green area) to reveal the complete extend of the binding site underneath. The central catalytic cysteine is shown in stick representation. Calpeptin (orange sticks) bound to CatL (c) and M^{pro} (d). Important substrate binding pockets are colored and labelled S1 to S4. Calpeptin derived from S-Calpeptin (teal sticks) bound to CatL (e) and M^{pro} (f), and GC-376 aldehyde derived from GC-376 (purple sticks) bound to CatL (g) and M^{pro} (h) in similar representation.

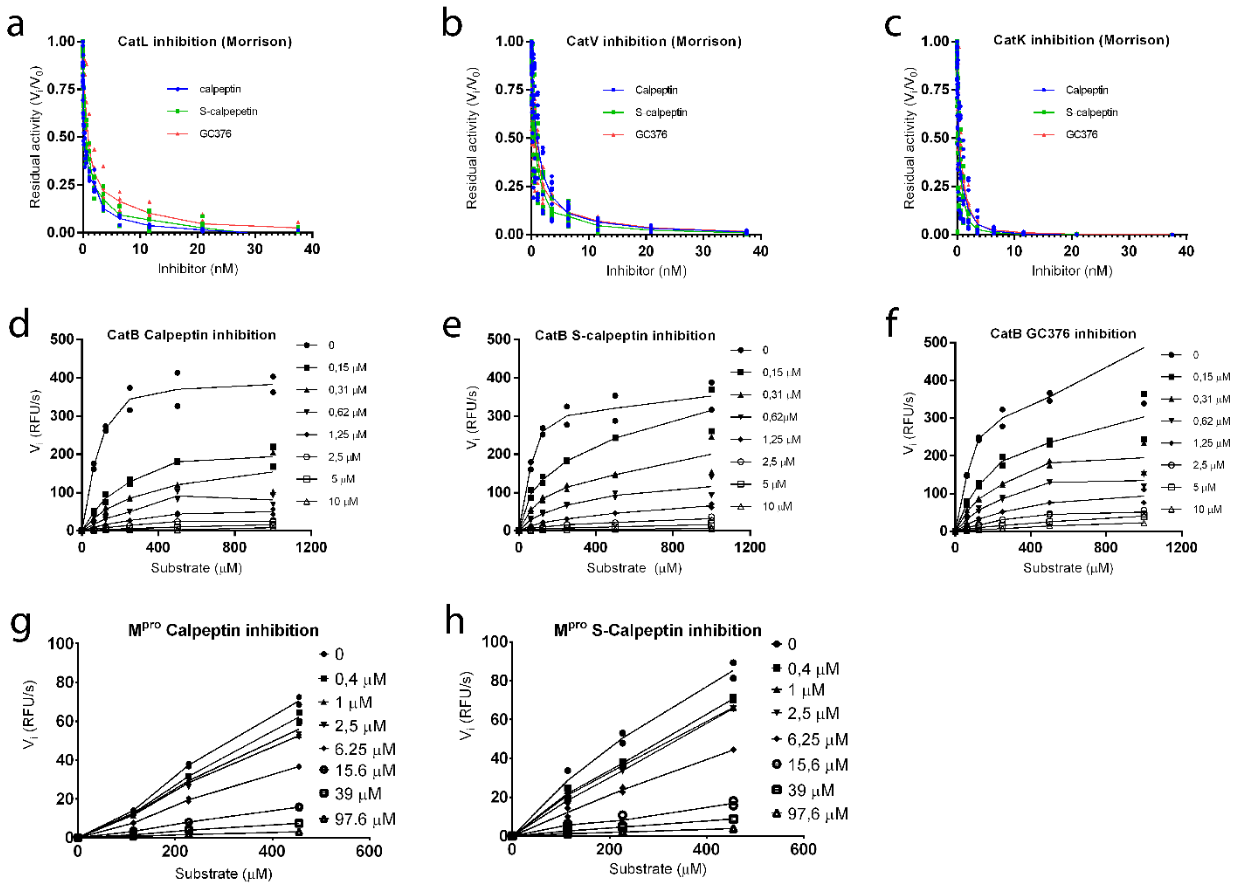

Figure S3.

Inhibition of human cathepsin and SARS-CoV-2 Mpro by Calpeptin and Calpeptin-like compounds (*Ki* determination). (a-c) Morrison inhibition plots of residual cathepsin activity against different inhibitor concentrations (standard errors are shown dotted lines). (d-h) Mixed-model inhibition plots of either cathepsin or M^{pro} reaction velocities (RFU/seconds) measured at several substrate concentrations for different inhibitor concentrations (standard errors are shown as bars).

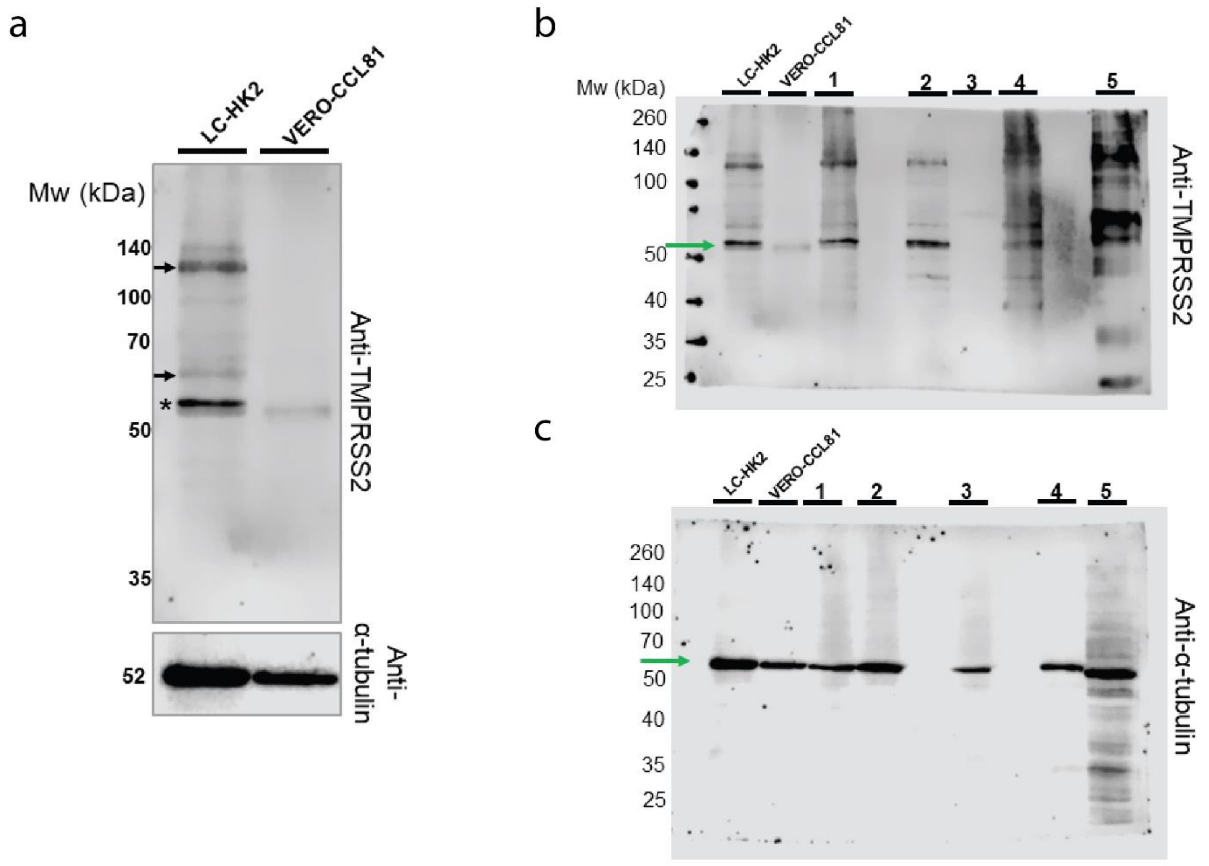

Figure S4.

Analysis of TMPRSS2 expression by Western blot. (a) LC-HK2 and VERO-CCL81 cells were lysed and subjected to SDS–PAGE following Western blot analysis under reducing conditions. TMPRSS2 was expressed at low levels in VERO-CCL81 and clearly expressed in LC-HK2 detectable as ~ 54-kDa band corresponding to the proenzyme form (zymogen) (*) and two additional major bands migrating at ~ 64-kDa and ~ 110-130 kDa (arrows). As loading control α-tubulin was used. (b-c) Uncropped images of source Western blots shown in (a). Wells 1 to 5 in (b-c) are not included in the manuscript experiments.

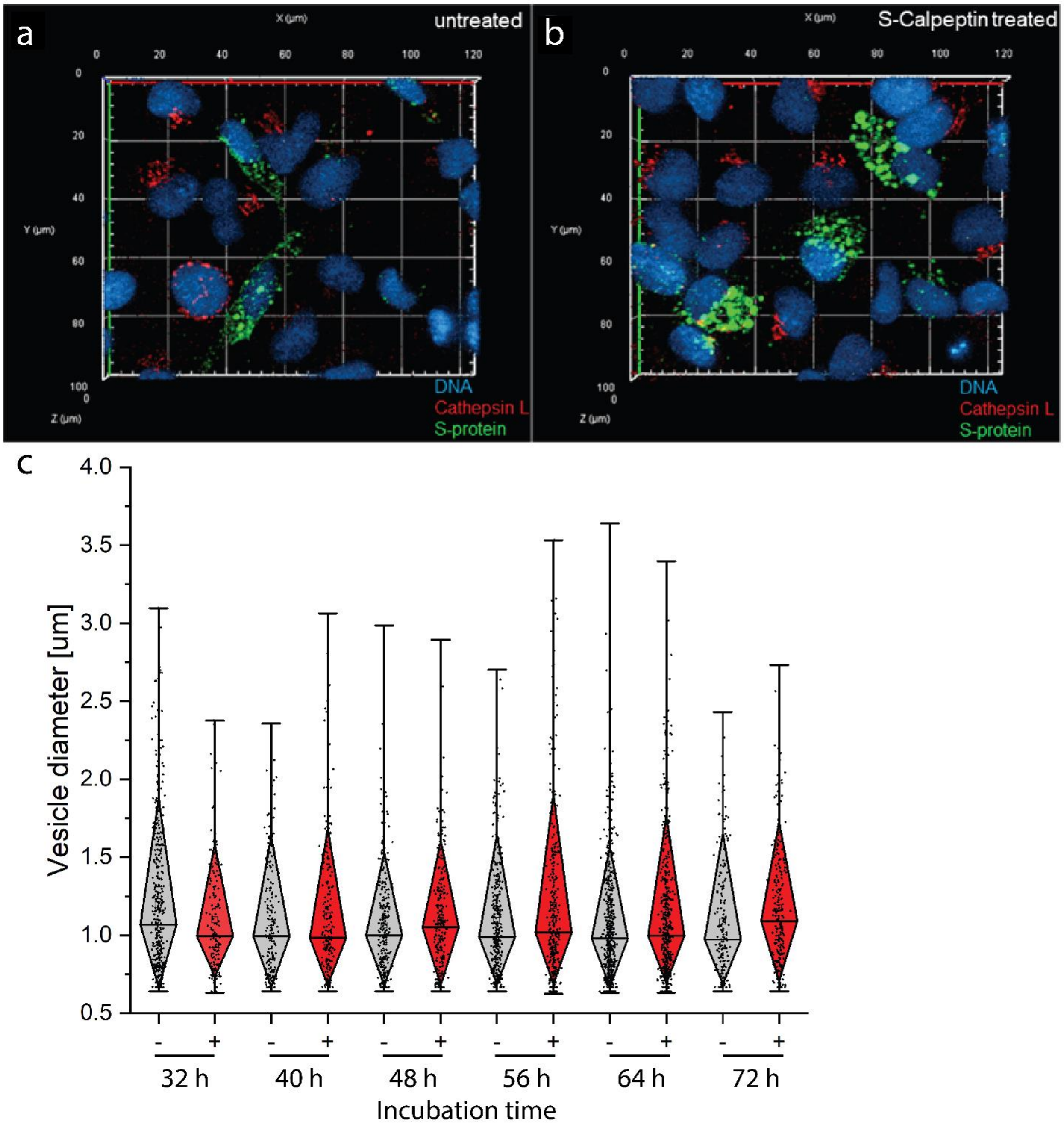

Figure S5.

Viral S-protein immunofluorescence microscopy. Representative image from 3D fluorescence microscopy of LC-HK2 cells infected with SARS-CoV-2 at a multiplicity of infection (M.O.I) of 0.05 (a) or treated with S-Calpeptin (b). Co-staining of S-protein (green), CatL (red) and DNA by Hoechst (blue). (c) Vesicle size analysis of various incubation timepoints after SARS-CoV-2 infections in the absence (-) or presence (+) of S-Calpeptin. Images were processed using ZEN (Blue) 2.6 software (ZEISS). Data are shown in diamond box plots (quartiles around median as diamond box, outer quartiles as whiskers).

208

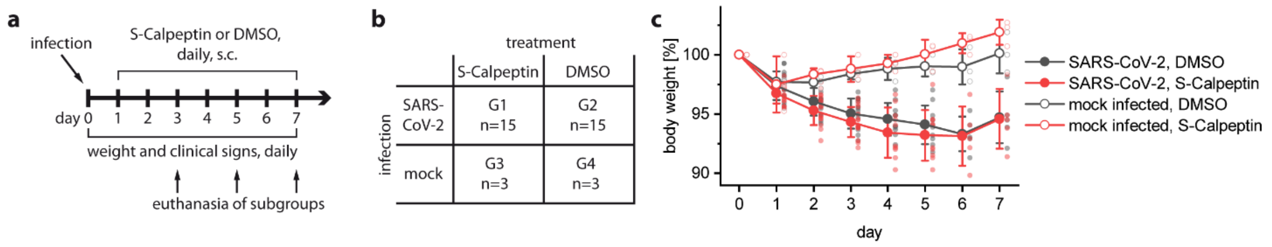

Figure S6.

Study design and weight of Golden Syrian hamsters infected with SARS-CoV-2 and treated with S-Calpeptin. (a) Study design. The experiment lasted for seven days, animals were infected with SARS-CoV-2 or non-infected (Dulbecco's Modified Eagle Medium with 2.5% fetal bovine serum) intranasally on day zero and treated daily for seven days with 1 mg/kg of S-Calpeptin or vehicle (DMSO) subcutaneously. Subgroups of SARS-CoV-2 infected animals (n=5, treated or not) were euthanized on days 3, 5 and 7 post-infection (p.i.). Non-infected animals (treated or not) were euthanized on day 7 p.i. (b) Overview over subgroups of all animals. G1: SARS-CoV-2-infected, treated with S-Calpeptin; G2: SARS-CoV-2-infected, injected with DMSO; G3: Non-infected, treated with S-Calpeptin; G4: Non-infected, injected with DMSO. (c) Mean percentual weight change from day of infection of all four experimental groups. Error bars represent standard deviation. Weight loss was significantly different between G1/G2 and G3/G4 ($p \leq 0.05$). Data from individual hamsters (smaller circles) are depicted with offset.

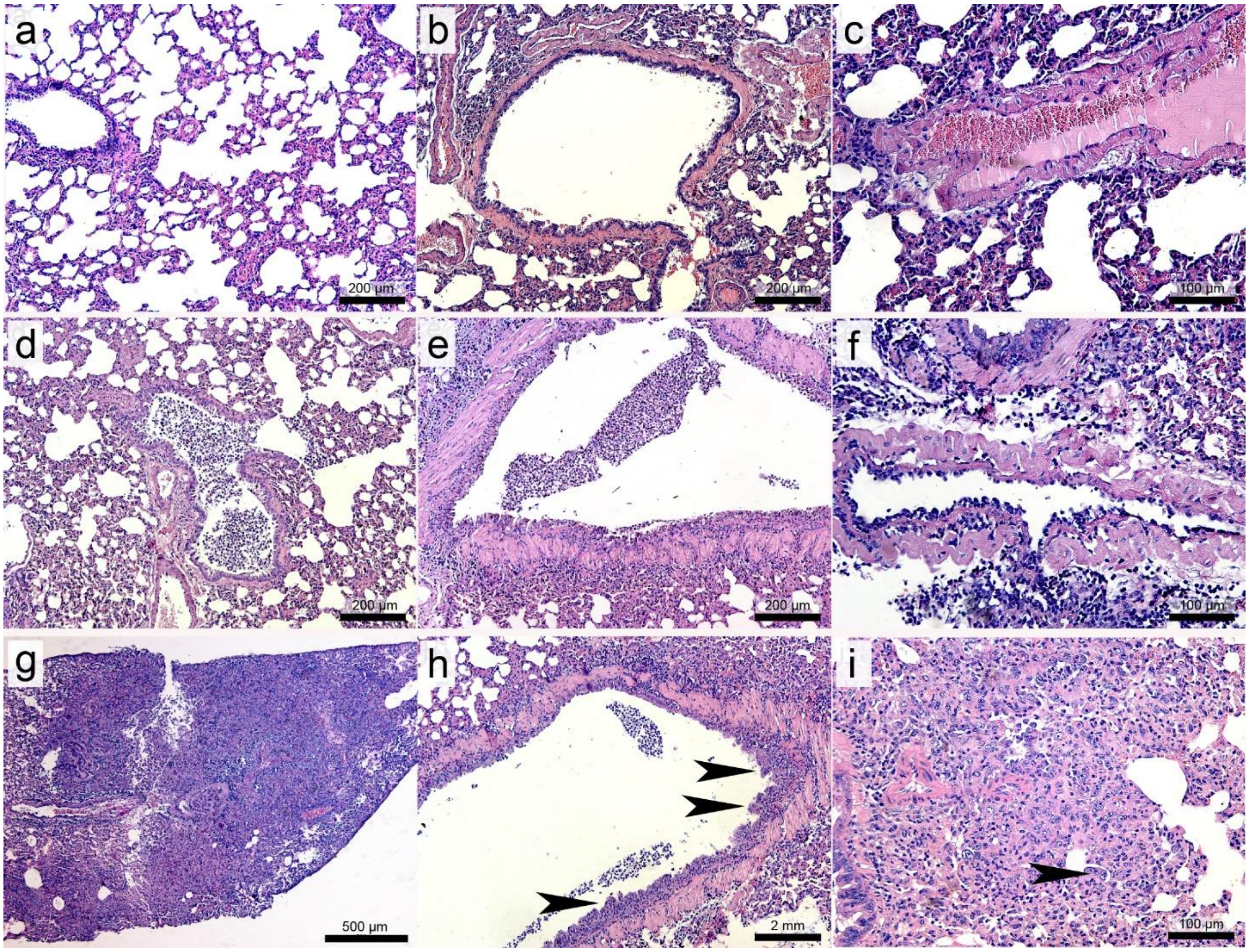

Figure S7.

Representative histopathology findings in the lungs of Golden Syrian hamsters, hematoxylin and eosin stain. (a) Uninfected control with normal alveolar septa. (b) Bronchus from an uninfected control. (c) Normal endothelial cells in an uninfected control. (d) Suppurative bronchitis in a SARS-CoV-2-infected hamster treated with 1:100 DMSO, day 3 post-infection (p.i.). (e) Necrosis of the bronchial epithelium and suppurative inflammation in a SARS-CoV-2-infected hamster treated with 1:100 DMSO, day 3 p.i. (f) Endothelialitis with hypertrophied endothelial cells and subendothelial inflammatory infiltrate in a medium sized blood vessel of a SARS-CoV-2-infected hamster treated with 1 mg/kg of Calpeptin, day 5 p.i. (g) Severe and diffuse bronchointerstitial pneumonia effacing the pulmonary parenchyma of a SARS-CoV-2-infected hamster treated with 1 mg/kg of Calpeptin, day 5 p.i. (h) The bronchial epithelium is

hyperplastic (arrows). (i) Alveolar type II epithelial cells are hyperplastic and line the alveoli with occasional atypical syncytial cells (arrow).

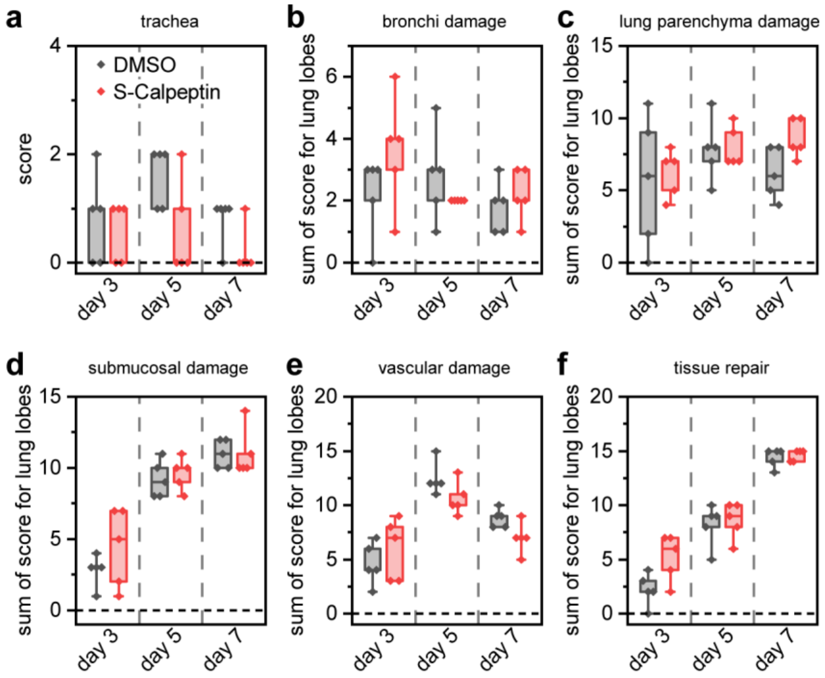

Figure S8.

Histopathological analysis of tissue from SARS-CoV-2 infected and treated hamsters.

Analysis for (a) trachea, (b) bronchi damage, (c) lung parenchyma damage, (d) submucosal damage, (e) vascular damage, and (f) tissue repair. For (b-f) score is sum of analysis of 5 lung lobes per animal. Values are expressed in median, interquartiles and range.

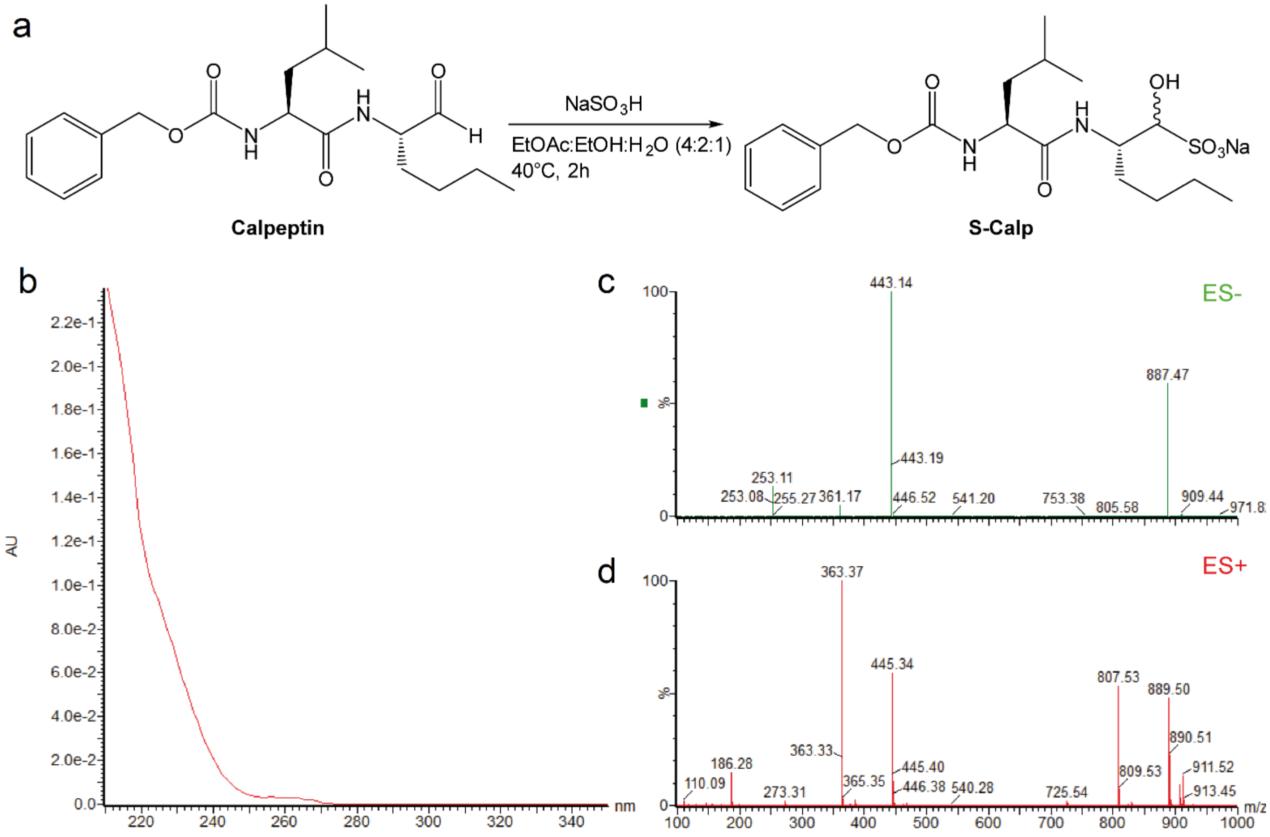

Figure S9.

Synthesis of S-Calpeptin from Calpeptin. (a) Reaction scheme to create S-Calpeptin, the bisulfite adduct of Calpeptin. (b) UV spectrum of product. (c) ES- and (d) ES+ spectrum of product. Calculated m/z for $C_{20}H_{31}N_4O_7S$ $[M-Na]^-$ is 443.1857 Da, found: 443.1887 Da.

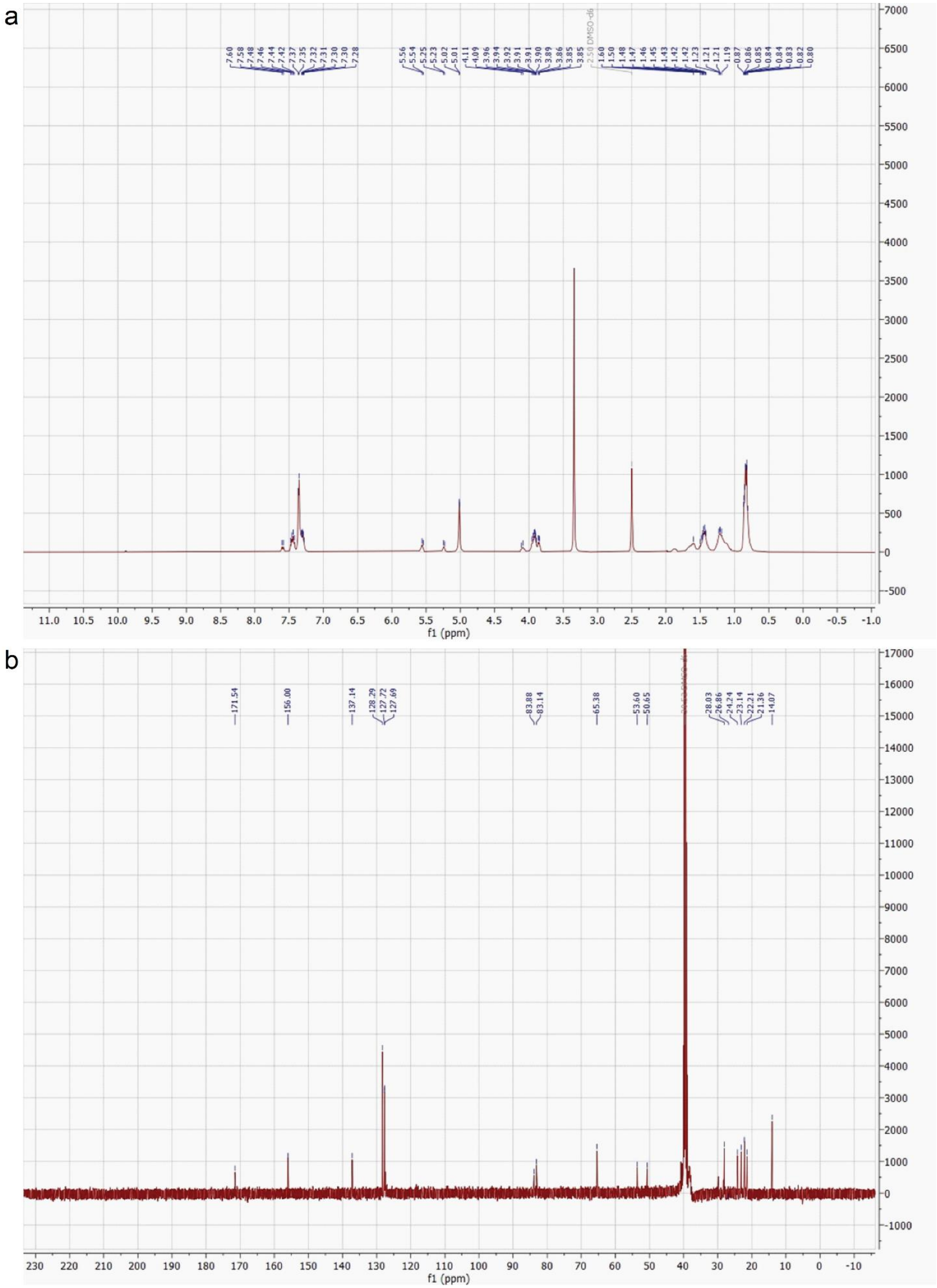
a
DMSO-d6
f1 (ppm)
b
171.54
156.00
137.14
128.29
127.72
127.69
83.88
83.14
65.38
53.60
50.65
28.03
26.86
24.24
23.14
22.21
21.36
14.07
f1 (ppm)

Figure S10.

NMR analysis of S-Calpeptin. (a) ^{1}H NMR of bisulfite adduct S-Calpeptin recorded at 400 MHz in DMSO-d_6. (b) ^{13}C NMR of bisulfite adduct S-Calpeptin recorded at 100 MHz in DMSO-d_6.

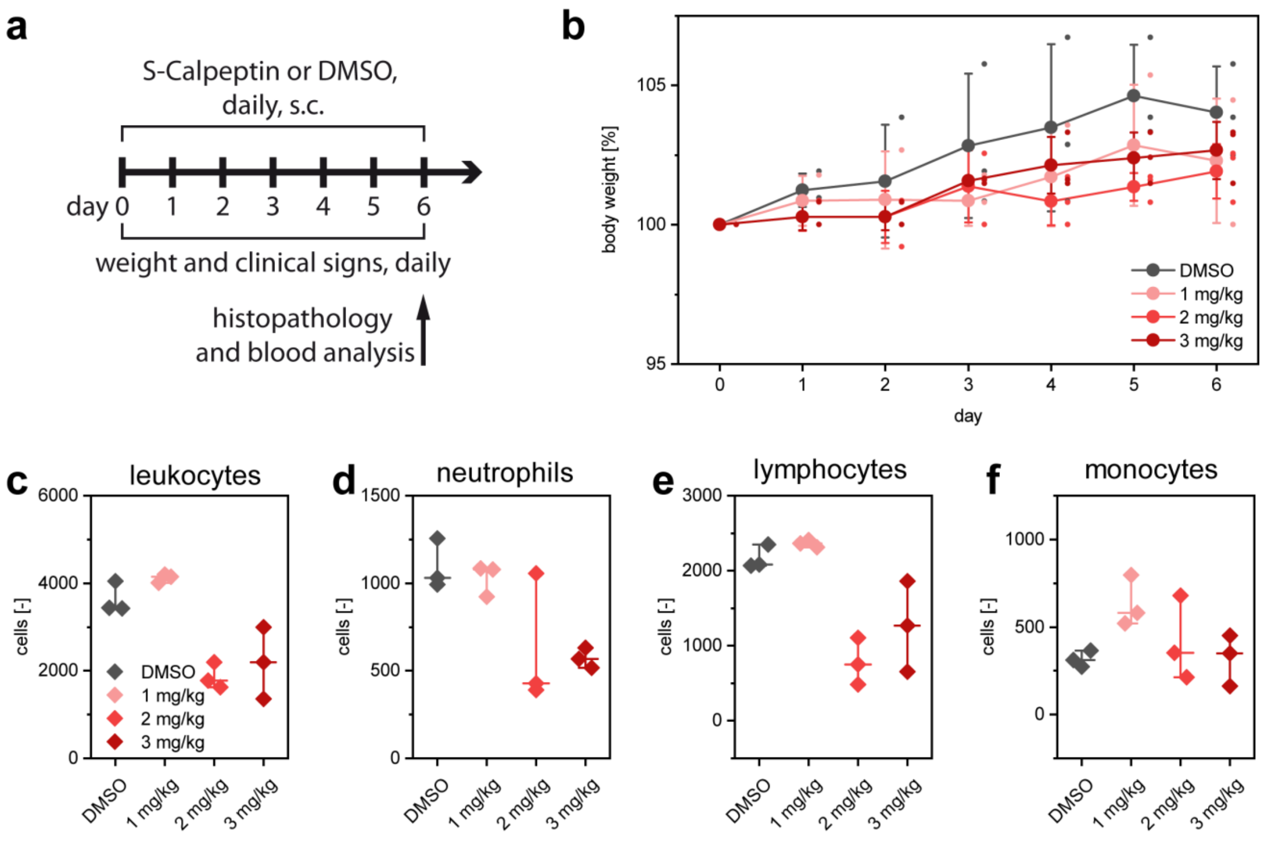

Figure S11.

S-Calpeptin toxicity experiment. (a) Study design for assessing toxicity of increasing S-Calpeptin doses. Each group included 3 Golden Syrian hamsters. Hamsters received a daily subcutaneous dose of S-Calpeptin (1 mg/kg, 2 mg/kg, or 3 mg/kg of body weight) suspended in 1:100 DMSO or a solution of 1:100 DMSO for seven consecutive days, starting at day zero. (b) Mean percentual weight change from day of infection of all four experimental groups. Data from individual hamsters (smaller circles) are depicted with an offset. Blood was analyzed on last day of experiment for cell count of leukocytes (c), neutrophils (d), lymphocytes (e), and monocytes (f). Median and range for all four groups is indicated.

Figure S12.

Structure cards, with detailed information about inhibitors derived from X-ray structures.

Cathepsin L, PDB: 7Z3T

Surface and cartoon depiction, superimposed monomers of AU

Superimposed cartoon:	Superimposed surface:
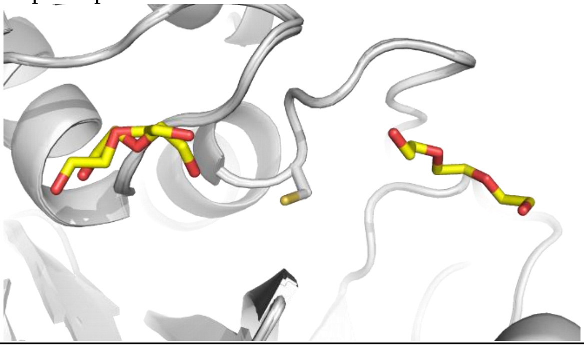	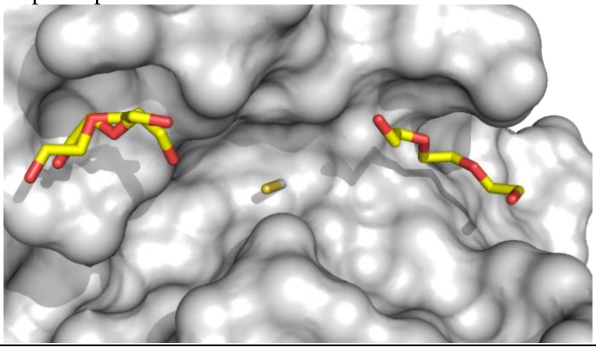

Structure and ligand features

**PDB structure code:** 7Z3T	**PDB Ligand code: -**
**Resolution:** 1.6 Å	**R_{work}/R_{free}:** 0.16/0.19
**Space group:** 1 (P1)	**Unit cell:** 56.94 Å, 62.14 Å, 67.47 Å, 105.17°, 94.23°, 115.58°
**Occupancy: -**	**Ligand RSCC/RSR: -**

Cathepsin L with bound Calpeptin, PDB: 7Z58

Molecule A: Refinement 2Fo-Fc map, 1.0 σ-level

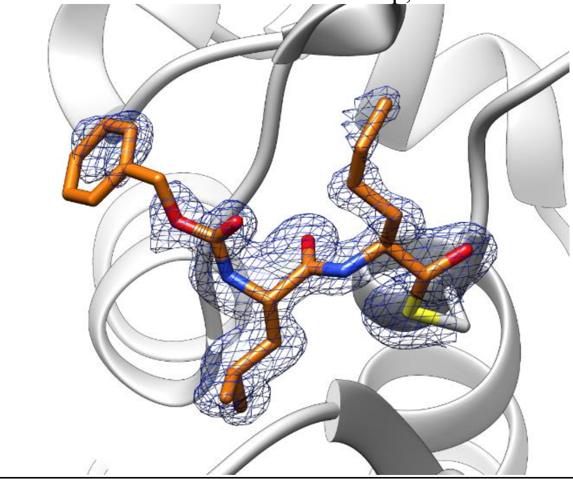

Molecule B: Refinement 2Fo-Fc map, 1.0 σ-level

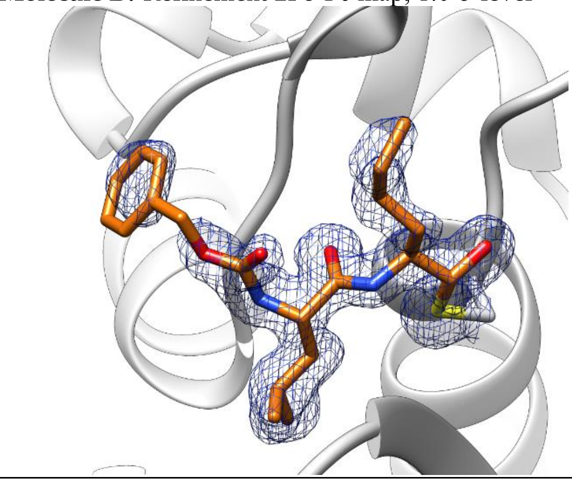

Molecule C: Refinement 2Fo-Fc map, 1.0 σ-level

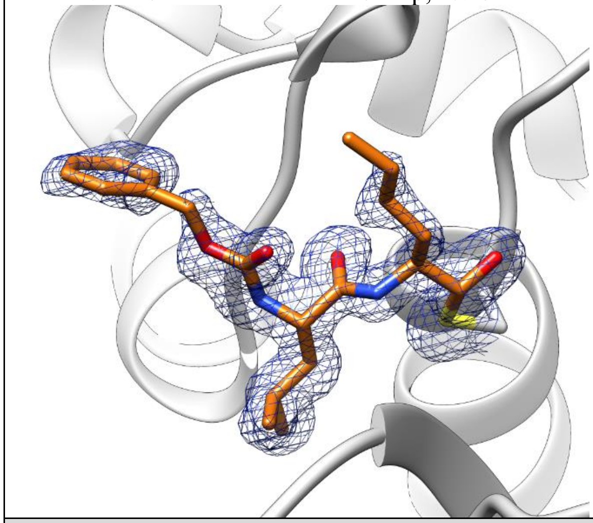

Molecule D: Refinement 2Fo-Fc map, 1.0 σ-level

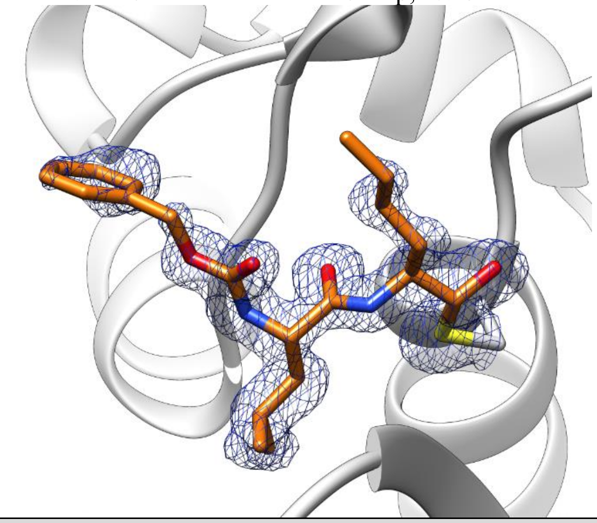

Surface and cartoon depiction, superimposed monomers of AU

Superimposed Cartoon:

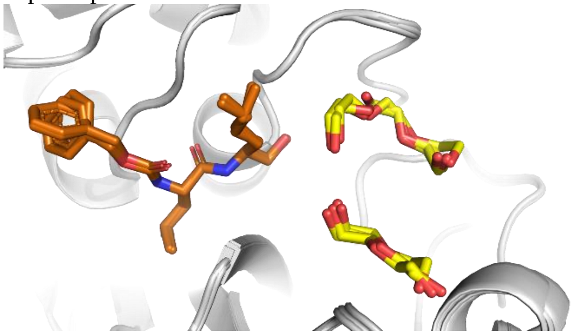

Superimposed surface:

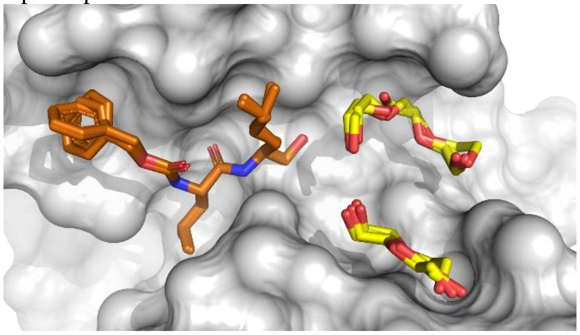

Structure and ligand features

**PDB structure code:** 7Z58	**PDB Ligand code:** RN2
**Resolution:** 1.35 Å	**R_{work}/R_{free}:** 0.13/0.16
**Space group:** 1 (P1)	**Unit cell:** 57.18 Å, 62.45 Å, 67.82 Å, 105.58°, 93.13°, 115.56°
**Occupancy:** 1.0, 1.0, 1.0, 1.0	**Ligand RSCC/RSR:** 0.85/0.23, 0.89/0.20, 0.89/0.17, 0.89/0.23

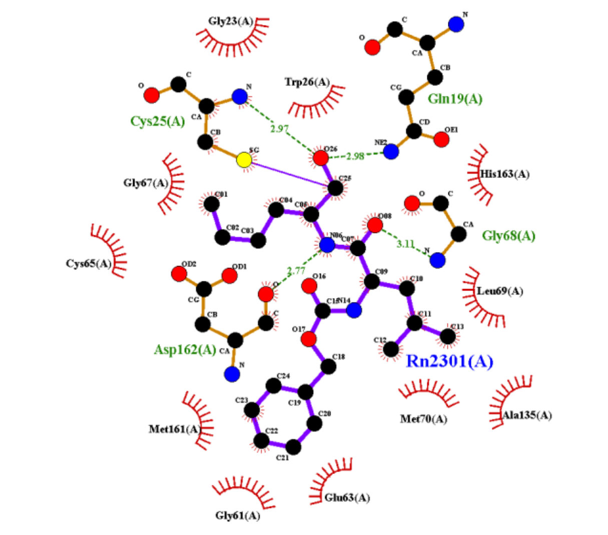	

Gly23(A)
Trp26(A)
Gln19(A)
Cys25(A)
His163(A)
Gly67(A)
Gly68(A)
Cys65(A)
Leu69(A)
Asp162(A)
Rn2301(A)
Met161(A)
Met70(A)
Ala135(A)
Glu63(A)
Gly61(A)
2.97
2.98
3.11
2.77

Cathepsin L with bound GC-376, PDB: 7QKB

Molecule A: Refinement 2Fo-Fc map, 1.0 σ-level

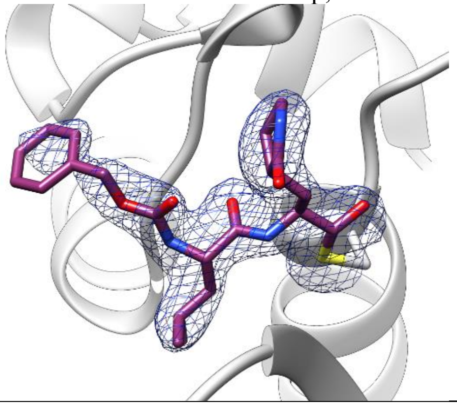

Molecule B: Refinement 2Fo-Fc map, 1.0 σ-level

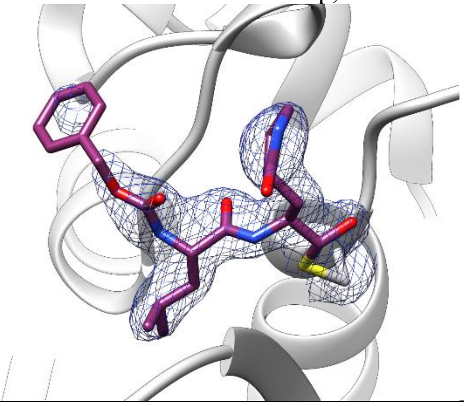

Molecule C: Refinement 2Fo-Fc map, 1.0 σ-level

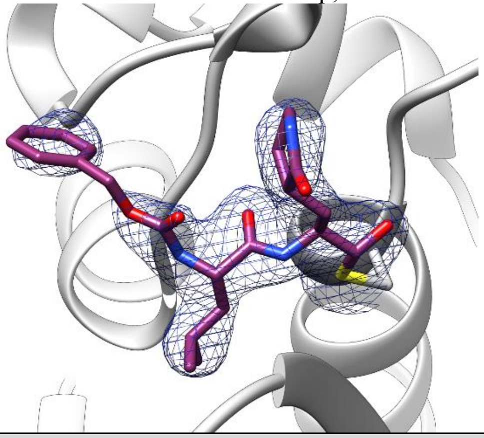

Molecule D: Refinement 2Fo-Fc map, 1.0 σ-level

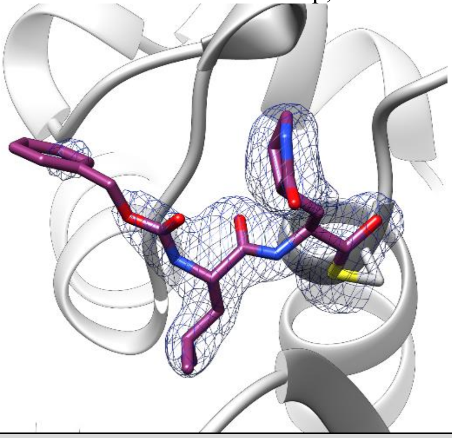

Surface and cartoon depiction, superimposed monomers of AU

Superimposed Cartoon:

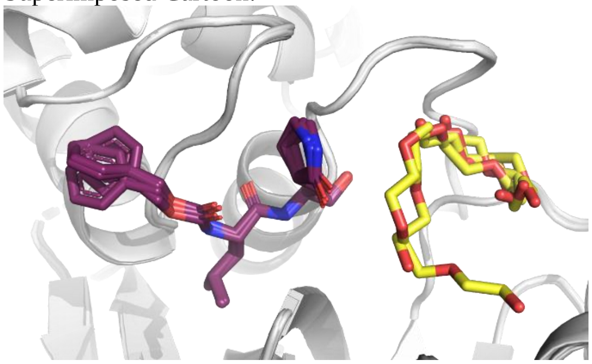

Superimposed surface:

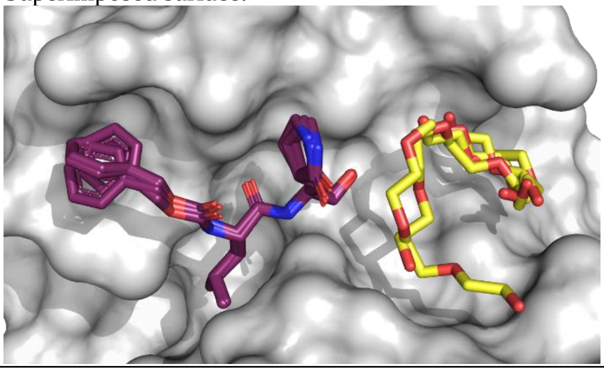

Structure and ligand features

**PDB structure code:** 7QKB	**PDB Ligand code:** UED
**Resolution:** 1.8 Å	**R_{work}/R_{free}:** 0.17/0.20
**Space group:** 1 (P1)	**Unit cell:** 57.29 Å, 62.45 Å, 67.82 Å, 105.31°, 93.52°, 115.93°
**Occupancy:** 1.0, 1.0, 1.0, 1.0 (A, B, C, D)	**Ligand RSCC/RSR:** 0.88/0.24, 0.89/0.19, 0.89/0.19, 0.90/0.26 (A, B, C, D)

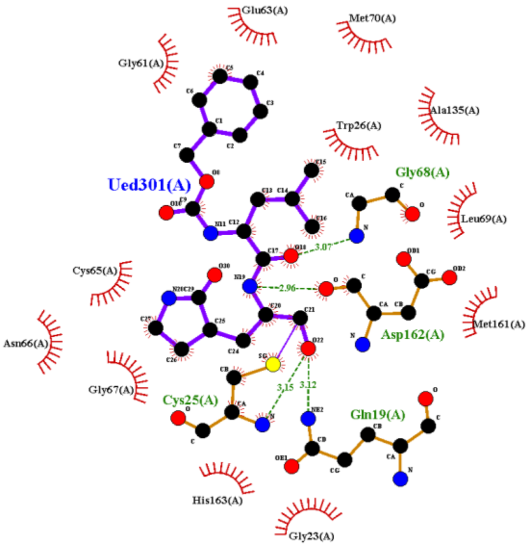
Glu63(A)
Met70(A)
Gly61(A)
Ala135(A)
Trp26(A)
Ued301(A)
Gly68(A)
Leu69(A)
3.07
Cys65(A)
2.96
Met161(A)
Asp162(A)
Asn66(A)
Gly67(A)
3.15
3.12
Cys25(A)
Gln19(A)
His163(A)
Gly23(A)

Cathepsin L with bound S-Calpeptin, PDB: 7QKC

Molecule A: Refinement 2Fo-Fc map, 1.0 σ-level

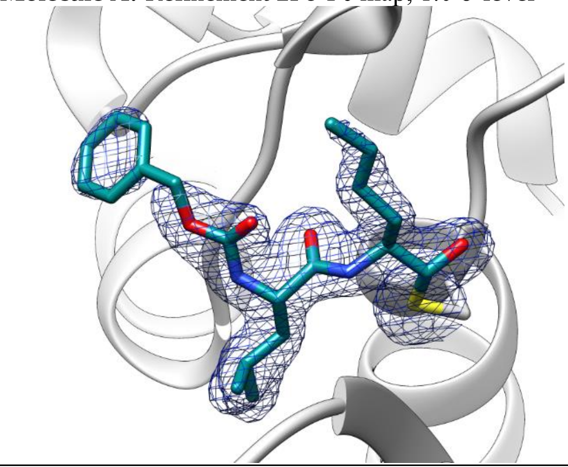

Molecule B: Refinement 2Fo-Fc map, 1.0 σ-level

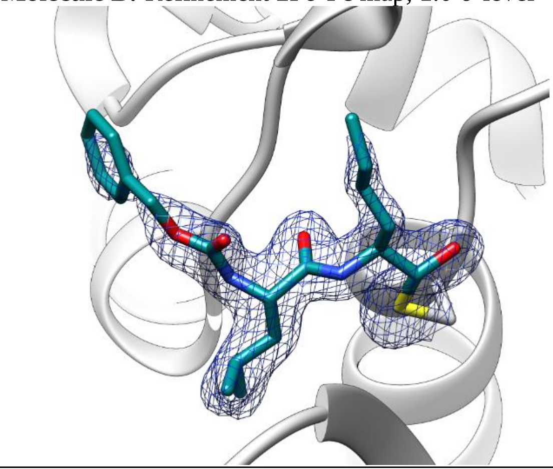

Molecule C: Refinement 2Fo-Fc map, 1.0 σ-level

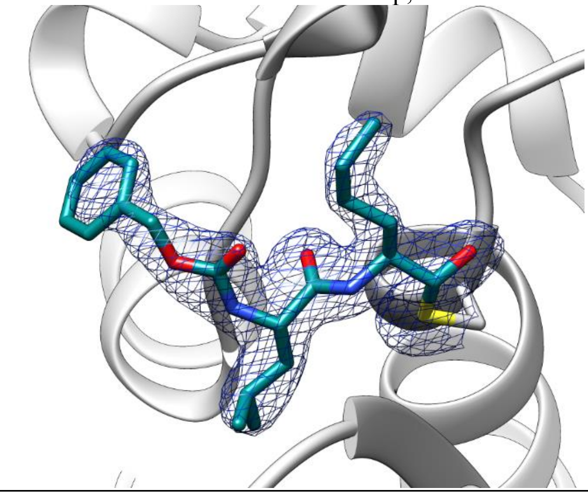

Molecule D: Refinement 2Fo-Fc map, 1.0 σ-level

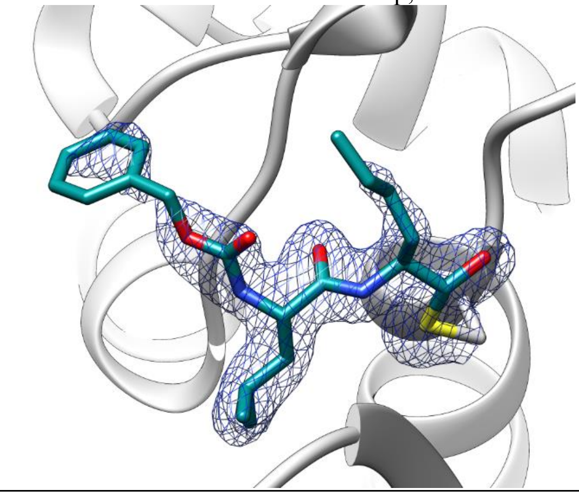

Surface and cartoon depiction, superimposed monomers of AU

Superimposed cartoon:

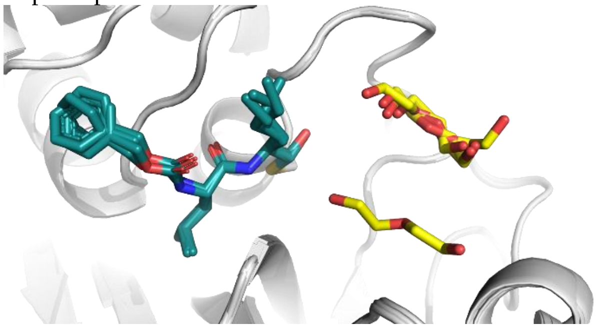

Superimposed surface:

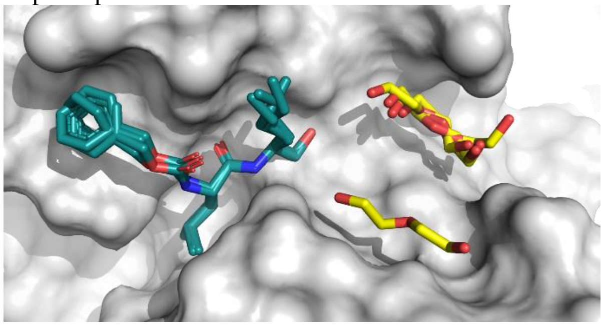

Structure and ligand features

**PDB structure code:** 7QKC	**PDB Ligand code:** RN2
**Resolution:** 1.69 Å	**R_{work}/R_{free}:** 0.15/0.18
**Space group:** 1 (P1)	**Unit cell:** 113.67 Å, 53.36 Å, 44.99 Å, 90.00°, 102.51°, 90.00°
**Occupancy:** 1.0, 1.0, 1.0, 1.0 (A, B, C, D)	**Ligand RSCC/RSR:** 0.80/0.27, 0.87/0.24, 0.90/0.22, 0.89/0.27 (A, B, C, D)

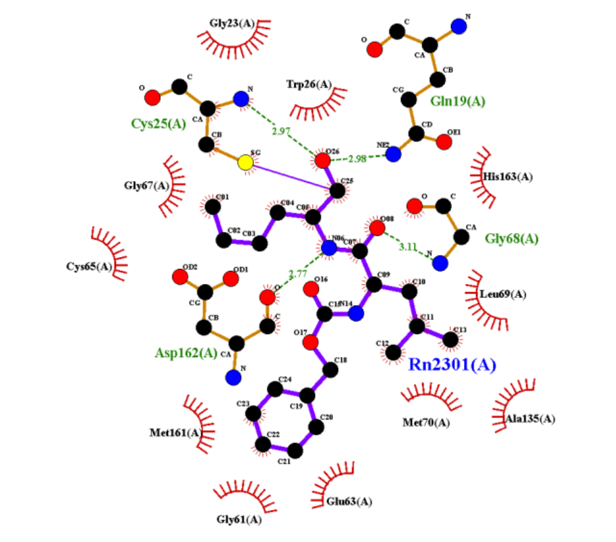
Gly23(A)
Trp26(A)
Gln19(A)
Cys25(A)
2.97
2.98
His163(A)
Gly67(A)
Gly68(A)
3.11
Cys65(A)
2.77
Leu69(A)
Asp162(A)
Rn2301(A)
Met161(A)
Met70(A)
Ala135(A)
Glu63(A)
Gly61(A)

M^{pro} with bound Calpeptin, PDB: 7AKU (Günther et al. 2021, Science)

Molecule A: Refinement 2Fo-Fc map, 1.0 σ-level

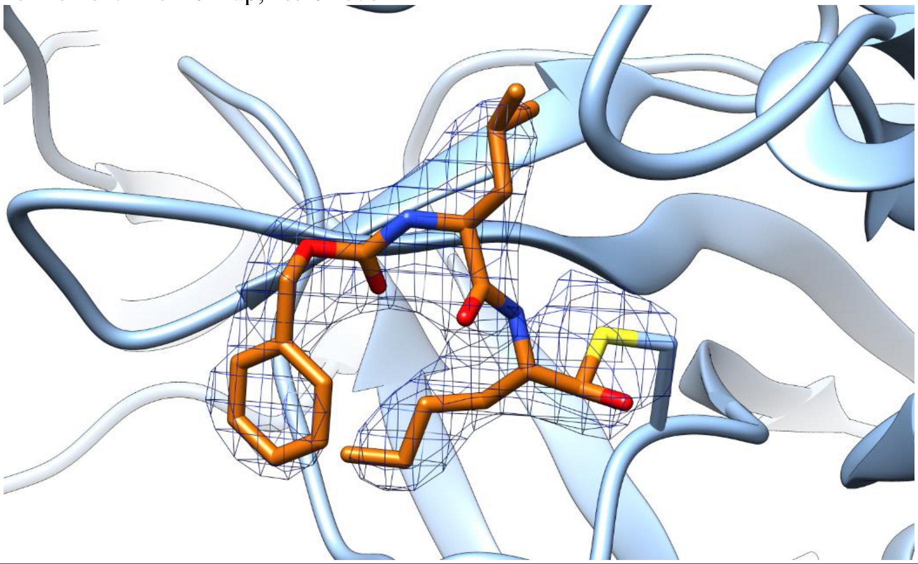

Surface and cartoon depiction

Cartoon:

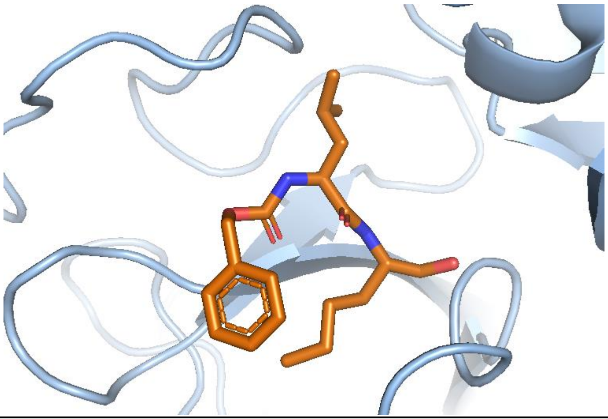

Surface:

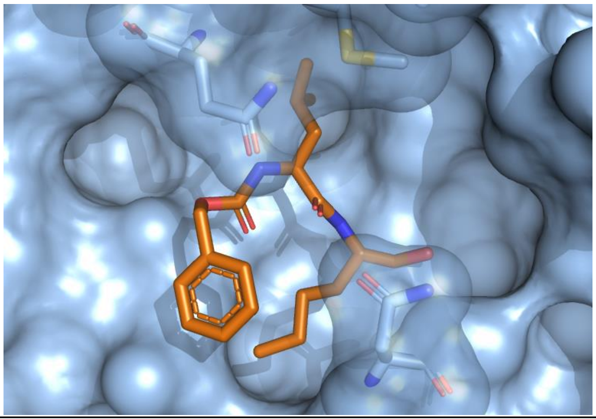

Structure and ligand features

**PDB structure code:** 7AKU	**PDB Ligand code:** RN2
**Resolution:** 2.50 Å	**R_{work}/R_{free}:** 0.18/0.24
**Space group:** 5 (C 1 2 1)	**Unit cell:** 114.67 Å, 53.84 Å, 45.12 Å, 90.00°, 101.86°, 90.00°
**Occupancy:** 1.0	**Ligand RSCC/RSR:** 0.93/0.17

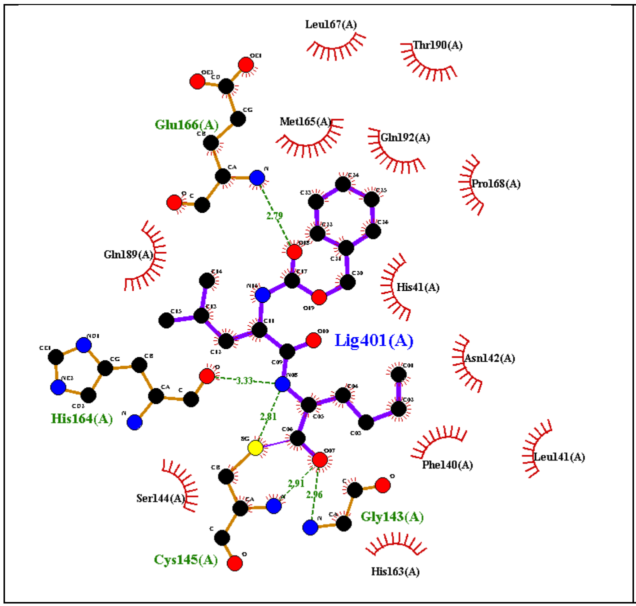
Leu167(A)
Thr190(A)
Glu166(A)
Met165(A)
Gln192(A)
Pro168(A)
Gln189(A)
His41(A)
Lig401(A)
Asn142(A)
His164(A)
Phe140(A)
Leu141(A)
Ser144(A)
Gly143(A)
Cys145(A)
His163(A)
2.79
3.33
2.81
2.91
2.96

280

M^{pro} with bound GC-376, PDB: 7QKA

Molecule A: Refinement 2Fo-Fc map, 1.0 σ-level

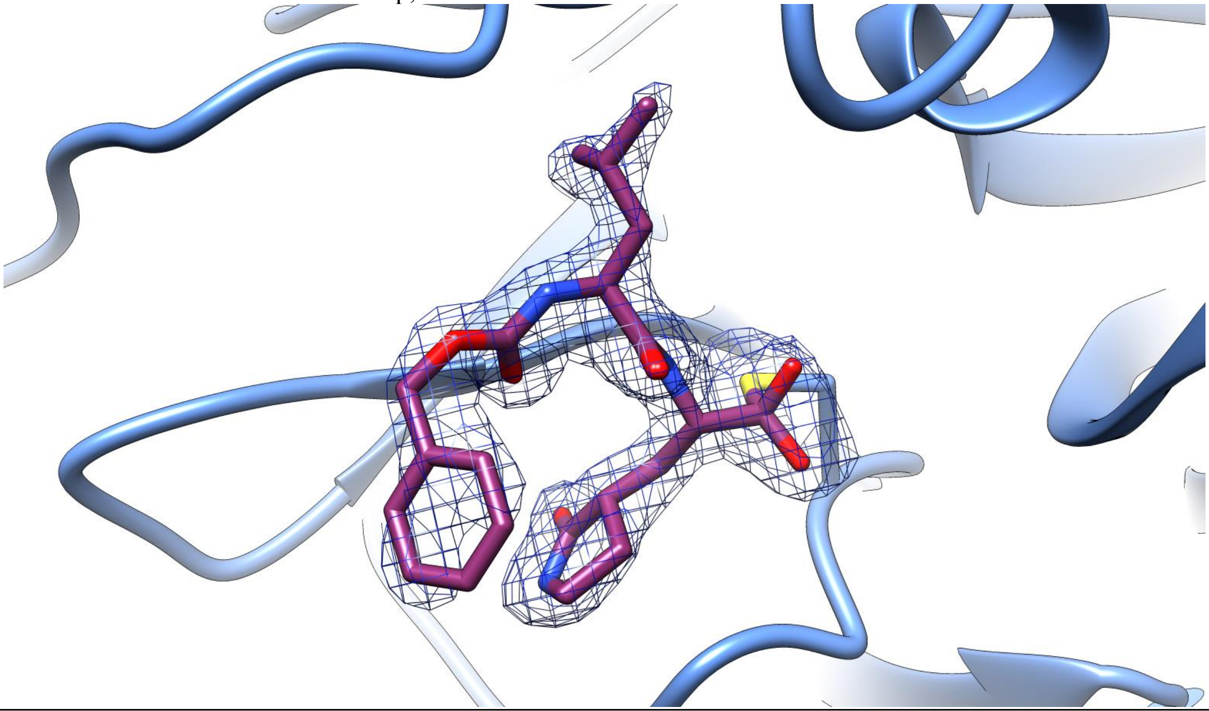

Surface and cartoon depiction

Cartoon:

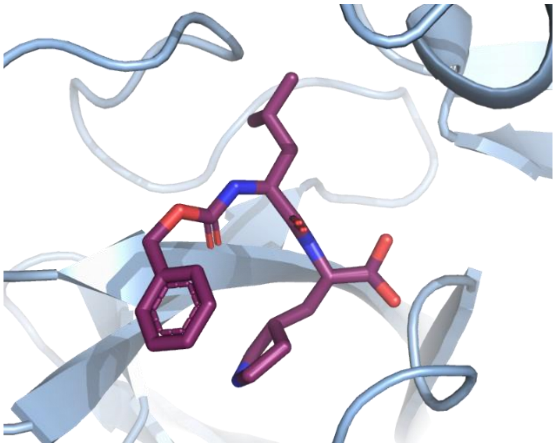

Surface:

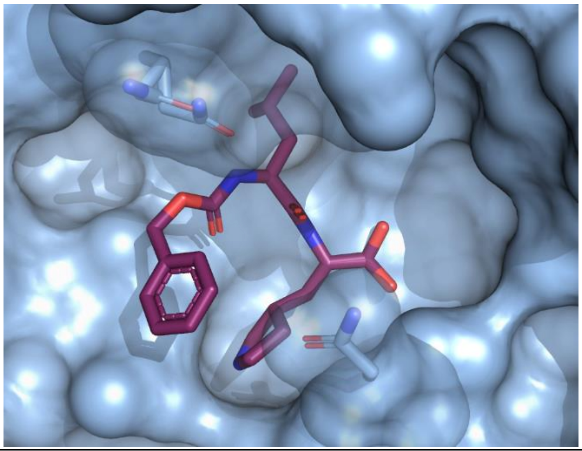

Structure and ligand features

**PDB structure code:** 7QKA	**PDB Ligand code:** UED
**Resolution:** 1.8 Å	**R_{work}/R_{free}:** 0.16/0.21
**Space group:** 5 (C 1 2 1)	**Unit cell:** 113.67 Å, 53.36 Å, 44.99 Å, 90.00°, 102.51°, 90.00°
**Occupancy:** 0.35 (conf A), 0.65 (conf B)	**Ligand RSCC/RSR:** 0.82/0.18 (conf A), 0.82/0.18 (conf B)

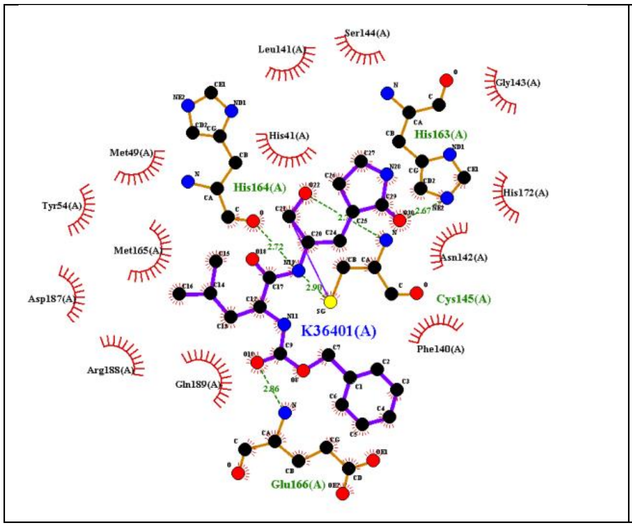
Ser144(A)
Leu141(A)
Gly143(A)
His41(A)
Met49(A)
His163(A)
His164(A)
His172(A)
Tyr54(A)
Met165(A)
Asn142(A)
Asp187(A)
Cys145(A)
K36401(A)
Phe140(A)
Arg188(A)
Gln189(A)
Glu166(A)
2.72
2.90
2.67
2.86

M^{pro} with bound S-Calpeptin, PDB: 7Z3U

Molecule A: Refinement 2Fo-Fc map, 0.5 σ-level

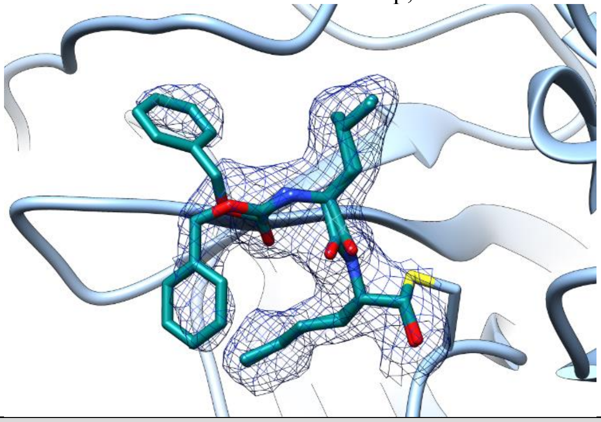

Molecule B: Refinement 2Fo-Fc map, 0.5 σ-level

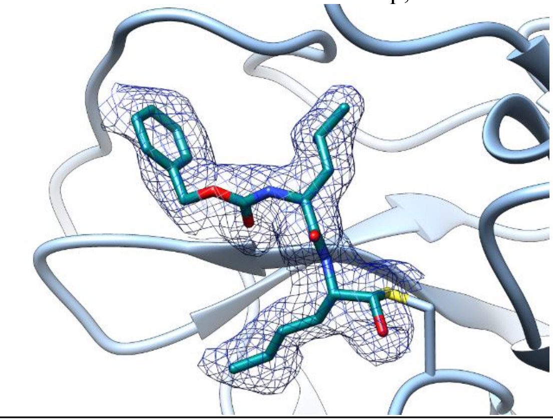

Surface and cartoon depiction, superimposed monomers of AU

Superimposed cartoon:

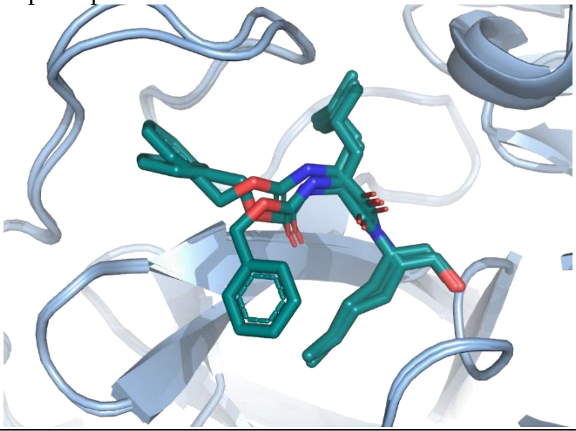

Superimposed surface:

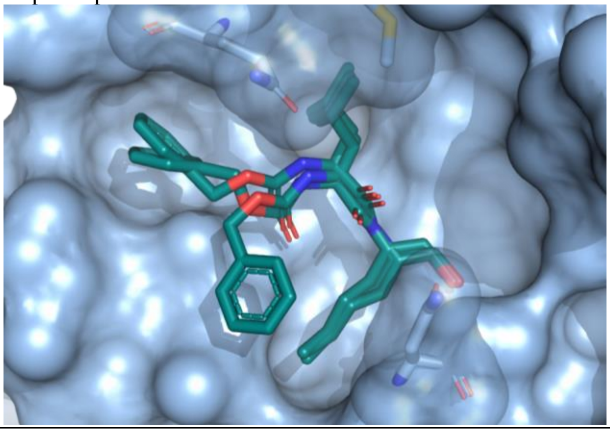

Structure and ligand features

**PDB structure code:** 7Z3U	**PDB Ligand code:** RN2
**Resolution:** 1.72 Å	**R_{work}/R_{free}:** 0.19/0.22
**Space group:** 19 (P 21 21 21)	**Unit cell:** 67.69 Å, 99.60 Å, 103.26 Å, 90.00°, 90.00°, 90.00°
**Occupancy:** 0.47 (AA), 0.53 (AB), 1.0 (B)	**Ligand RSCC/RSR:** 0.86/0.17, 0.86/0.17 (A, B)

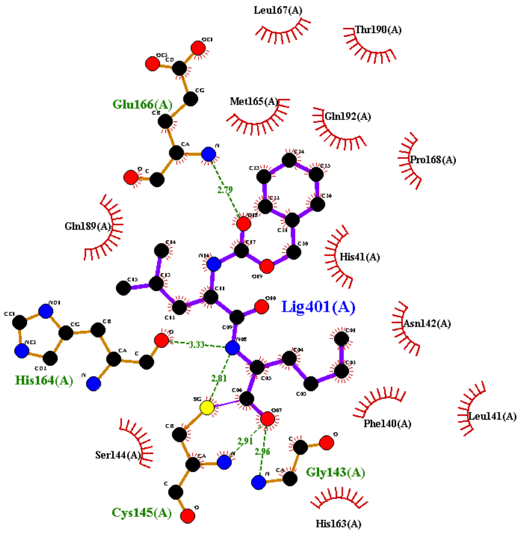

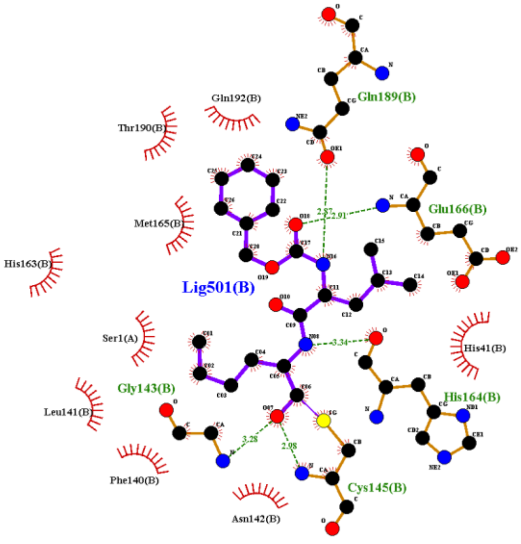

Cathepsin V with bound S-Calpeptin, PDB: 7QGW

**Molecule A:** Refinement 2Fo-Fc map, 1.0 σ-level	**Molecule B:** Refinement 2Fo-Fc map, 1.0 σ-level
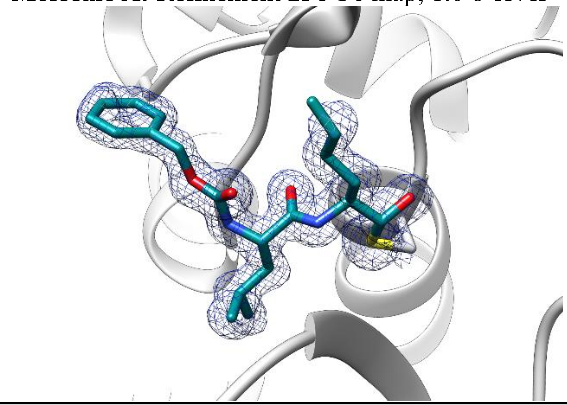	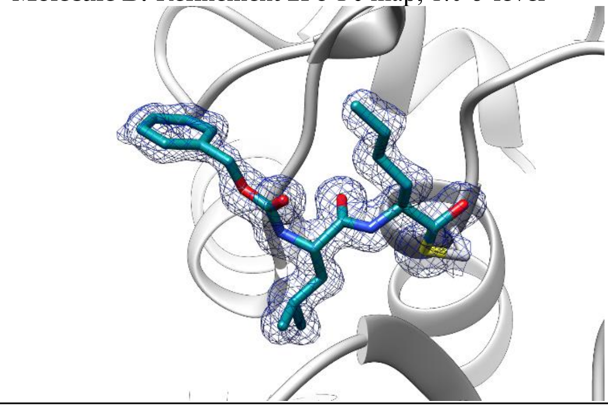

Surface and cartoon depiction, superimposed monomers of AU

Superimposed Cartoon:	Superimposed surface:
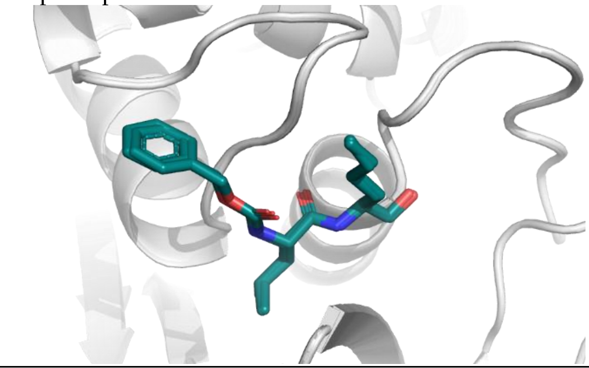	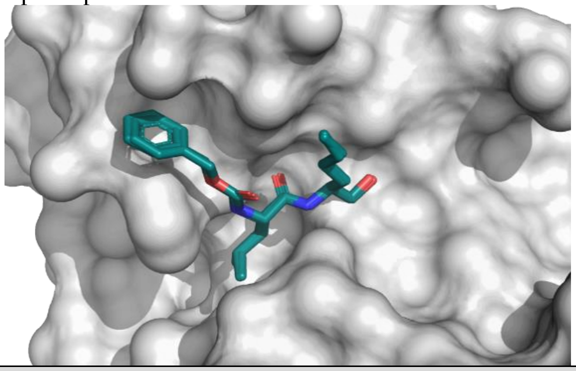

Structure and ligand features

**PDB structure code:** 7QGW	**PDB Ligand code:** RN2
**Resolution:** 1.3 Å	**R_{work}/R_{kick}:** 0.17/0.20
**Space group:** 96 (P 43 21 2)	**Unit cell:** 94.24 Å, 94.24 Å, 126.96 Å, 90.0°, 90.0°, 90.0°
**Occupancy:** 1.0, 1.0 (A, B)	**Ligand RSCC/RSR:** 0.92/0.09, 0.93/0.09 (A, B)
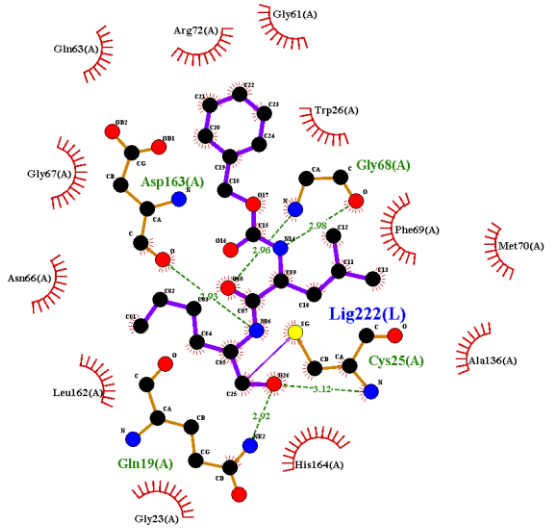	

Cathepsin K with bound S-Calpeptin (activated), PDB: 8C3D

Molecule A: Refinement 2Fo-Fc map, 1.0 σ-level

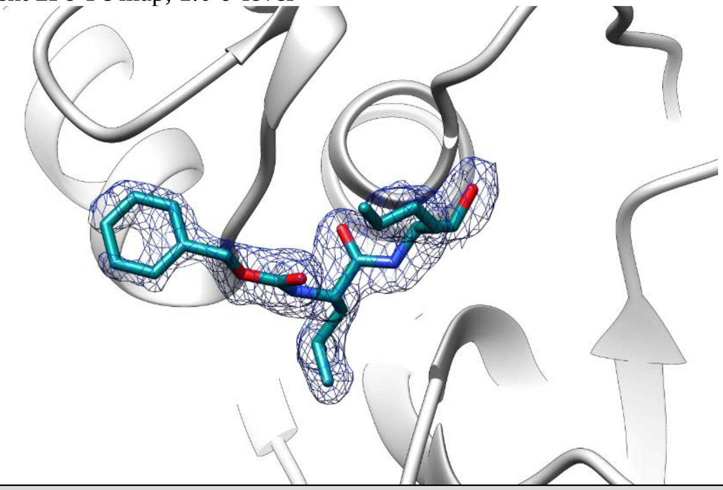

Surface and cartoon depiction

Superimposed Cartoon:	Superimposed surface:
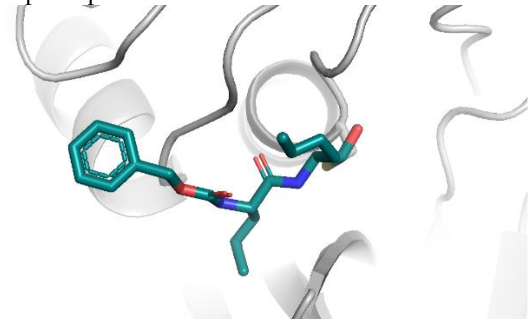	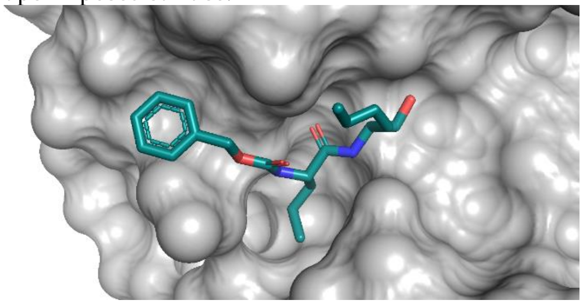

Structure and ligand features

**PDB structure code:** 7QGW	**PDB Ligand code:** RN2
**Resolution:** 1.3 Å	**R_{work}/R_{kick}:** 0.17/0.20
**Space group:** 96 (P 43 21 2)	**Unit cell:** 94.24 Å, 94.24 Å, 126.96 Å, 90.0°, 90.0°, 90.0°
**Occupancy:** 1.0, 1.0 (A, B)	**Ligand RSCC/RSR:** 0.92/0.09, 0.93/0.09 (A, B)

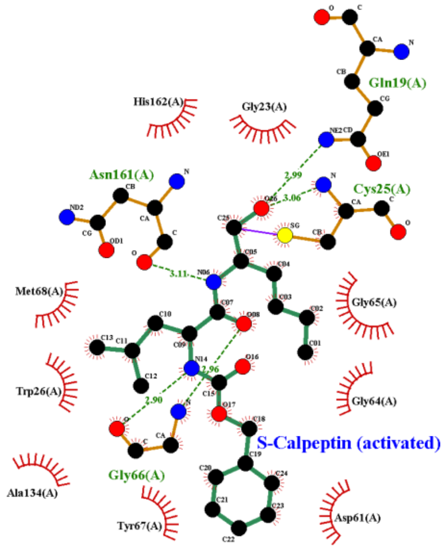

307

Table S1.

Score system for histopathological analysis. Parameters and scores for semiquantitative histopathological evaluation of lungs from hamsters experimentally infected with SARS-CoV-2.

**Parameter**	**Scores**	
	Bronchi	***Pulmonary parenchyma***
**Epithelial damage**	0 - normal<br>1 - degeneration (cuboidal cells)<br>2 - epithelial detachment<br>3 - necrosis and sloughing	0 - normal<br>1 - degeneration (cuboidal cells) and/or peribronchiolar inflammatory infiltrate<br>2 - epithelial detachment<br>3 - necrosis and sloughing and/or bronchiolar inflammatory infiltrate
**Submucosal damage**	0 - normal<br>1 - edema, vascular ectasia, or mild inflammatory infiltrate affecting 10-33% of the examined area<br>2 - moderate inflammatory infiltrate affecting 34-66% of the examined area<br>3 - marked inflammatory infiltrate affecting >67% of the examined area	
**Vascular damage**	0 - normal<br>1 - perivascular edema and/or endothelial hypertrophy<br>2 - mild perivascular inflammatory infiltrate and/or endothelialitis<br>3 - inflammatory infiltrate in the vascular wall and/or below the endothelium (vasculitis)	
**Tissue repair**	0 - normal<br>1 - repair with pre-ciliated cells and/or hyperplasia of non-ciliated epithelium<br>2 - hyperplasia of alveolar type II epithelial cells with or without atypia<br>3 – hyperplasia of goblet cells, and/or presence of granulation tissue and/or fibrosis	
