# Supplementary material for: Calpeptin is a potent cathepsin inhibitor and drug candidate for SARS-CoV-2 infections
Source: Commun Biol. 2023 Oct 18;6:1058. doi: 10.1038/s42003-023-05317-9 (PMC10584882; doi:10.1038/s42003-023-05317-9)
Supplement: Supplementary file 3 — Description of Additional Supplementary Files [file 42003_2023_5317_MOESM3_ESM.pdf]

Description of Additional Supplementary Files

File name: Supplementary Data 1
Description: Source data for the graphs in the figures.
