# Supplementary material for: Calpeptin is a potent cathepsin inhibitor and drug candidate for SARS-CoV-2 infections
Source: Commun Biol. 2023 Oct 18;6:1058. doi: 10.1038/s42003-023-05317-9 (PMC10584882; doi:10.1038/s42003-023-05317-9)
Supplement: Supplementary file 5 — Reporting Summary [file 42003_2023_5317_MOESM5_ESM.pdf]

nature portfolio

Corresponding author(s): D. Turk, C. Wrenger, A. Meents

Last updated by author(s): Jun 6, 2023

Reporting Summary

Nature Portfolio wishes to improve the reproducibility of the work that we publish. This form provides structure for consistency and transparency in reporting. For further information on Nature Portfolio policies, see our Editorial Policies and the Editorial Policy Checklist.

Statistics

For all statistical analyses, confirm that the following items are present in the figure legend, table legend, main text, or Methods section.

n/a	Confirmed	
☐	☒	The exact sample size (*n*) for each experimental group/condition, given as a discrete number and unit of measurement
☐	☒	A statement on whether measurements were taken from distinct samples or whether the same sample was measured repeatedly
☐	☒	The statistical test(s) used AND whether they are one- or two-sided<br>*Only common tests should be described solely by name; describe more complex techniques in the Methods section.*
☐	☒	A description of all covariates tested
☐	☒	A description of any assumptions or corrections, such as tests of normality and adjustment for multiple comparisons
☐	☒	A full description of the statistical parameters including central tendency (e.g. means) or other basic estimates (e.g. regression coefficient) AND variation (e.g. standard deviation) or associated estimates of uncertainty (e.g. confidence intervals)
☐	☒	For null hypothesis testing, the test statistic (e.g. *F*, *t*, *r*) with confidence intervals, effect sizes, degrees of freedom and *P* value noted<br>*Give P values as exact values whenever suitable.*
☐	☒	For Bayesian analysis, information on the choice of priors and Markov chain Monte Carlo settings
☐	☒	For hierarchical and complex designs, identification of the appropriate level for tests and full reporting of outcomes
☐	☒	Estimates of effect sizes (e.g. Cohen's *d*, Pearson's *r*), indicating how they were calculated

Our web collection on statistics for biologists contains articles on many of the points above.

Software and code

Policy information about availability of computer code

Data collection	LI-COR Odyssey Imaging system (model 2802) software, InuCyte 2020C Rev1 software
Data analysis	GraphPad Prism 8.0 & 9.1.1 software, ImageJ FIJI 2.9, Zen 2.6 blue ZEISS software, OriginLab Origin 2021b, XDS, COOT 0.9.8, MAIN, PyMOL 2.5, Chimera 1.14

For manuscripts utilizing custom algorithms or software that are central to the research but not yet described in published literature, software must be made available to editors and reviewers. We strongly encourage code deposition in a community repository (e.g. GitHub). See the Nature Portfolio guidelines for submitting code & software for further information.

Data

Policy information about availability of data

All manuscripts must include a data availability statement. This statement should provide the following information, where applicable:

- Accession codes, unique identifiers, or web links for publicly available datasets
- A description of any restrictions on data availability
- For clinical datasets or third party data, please ensure that the statement adheres to our policy

PDB: 7Z58, 7QKB, 7Z3U, 7QKA, 7QGW, 8C3D

Research involving human participants, their data, or biological material

Policy information about studies with human participants or human data. See also policy information about sex, gender (identity/presentation), and sexual orientation and race, ethnicity and racism.

Reporting on sex and gender	Not applicable
Reporting on race, ethnicity, or other socially relevant groupings	Not applicable
Population characteristics	Not applicable
Recruitment	Not applicable
Ethics oversight	Not applicable

Note that full information on the approval of the study protocol must also be provided in the manuscript.

Field-specific reporting

Please select the one below that is the best fit for your research. If you are not sure, read the appropriate sections before making your selection.

☒ Life sciences ☐ Behavioural & social sciences ☐ Ecological, evolutionary & environmental sciences

For a reference copy of the document with all sections, see nature.com/documents/nr-reporting-summary-flat.pdf

Life sciences study design

All studies must disclose on these points even when the disclosure is negative.

Sample size	We used at least triplicate measurements.
Data exclusions	For vesicle analysis data was excluded due to the image resolution limit.
Replication	Two independent experiments were performed for cell assay experiments
Randomization	Randomization was not performed during the animal experiments because groups were homogenized by weight. However, when animals of similar weight were detected, then they were chosen randomly to allocate to different groups. No other animal characteristic was considered besides weight to allocate animals in different groups.
Blinding	For the experiments performed in this study, blinding was relevant and performed during the histopathology analysis of the tissue samples collected from the animals. The board-certified pathologist was blind to the group assignment of the samples.

Reporting for specific materials, systems and methods

We require information from authors about some types of materials, experimental systems and methods used in many studies. Here, indicate whether each material, system or method listed is relevant to your study. If you are not sure if a list item applies to your research, read the appropriate section before selecting a response.

Materials & experimental systems

n/a	Involved in the study	
☐	☒	Antibodies
☐	☒	Eukaryotic cell lines
☒	☐	Palaeontology and archaeology
☐	☒	Animals and other organisms
☒	☐	Clinical data
☒	☐	Dual use research of concern
☒	☐	Plants

Methods

n/a	Involved in the study	
☒	☐	ChIP-seq
☒	☐	Flow cytometry
☒	☐	MRI-based neuroimaging

Antibodies

Antibodies used	TMPRSS2 rabbit antibody (Sigma-Aldrich; ZRB1633), horseradish peroxidase-conjugated anti-rabbit (Cell Signaling; 7074S), HRP-

Antibodies used	conjugated anti-mouse (KPL - SeraCare; 041806), anti-α-tubulin (Thermo Fisher Scientific; MA1-19401)
Validation	*Describe the validation of each primary antibody for the species and application, noting any validation statements on the manufacturer's website, relevant citations, antibody profiles in online databases, or data provided in the manuscript.*

Eukaryotic cell lines

Policy information about cell lines and Sex and Gender in Research

Cell line source(s)	VERO-CCL81 cell was initiated from the kidney tissue derived from a normal, adult African green monkey Cercopithecus aethiops, obtained from ATCC (ATCC® CCL-81). LC-HK2 cell line was derived from a tumor induced in nude rat by the inoculation of LC-HK1 cells which was spontaneously established from an explant of a cervical human non-small cell lung cancer metastasis. The cellular and biochemical characterization of LC-HK2 in reported in: Bonaldo, M. de F. et al. Comparative characterization of a human large cell lung carcinoma cell line and the xenograft derived cell line. Cell Biol Int Rep 15, 229–241 (1991); Manelli-Oliveira, R. & Machado-Santelli, G. M. Cytoskeletal and nuclear alterations in human lung tumor cells: a confocal microscope study. Histochem Cell Biol 115, 403–411 (2001); Cortez, B. A., Rezende-Teixeira, P., Redick, S., Doxsey, S. & Machado-Santelli, G. M. Multipolar mitosis and aneuploidy after chrysotile treatment: a consequence of abscission failure and cytokinesis regression. Oncotarget 7, 8979–8992 (2016).
Authentication	VERO-CCL81 was authenticated via ATCC and LC-HK2 was not authenticated
Mycoplasma contamination	VERO-CCL81 and LC-HK2 were regularly analyzed for mycoplasma via PCR reaction and mycoplasma contamination was not detected.
Commonly misidentified lines (See ICLAC register)	*Name any commonly misidentified cell lines used in the study and provide a rationale for their use.*

Animals and other research organisms

Policy information about studies involving animals; ARRIVE guidelines recommended for reporting animal research, and Sex and Gender in Research

Laboratory animals	Mesocricetus auratus, 6-8 weeks old, male
Wild animals	Not applicable
Reporting on sex	Only males were used, thus, results are only applicable to male sex.
Field-collected samples	Not applicable
Ethics oversight	All procedures were approved by the Committee on Animal Use and Experimentation from the Institute of Biomedical Sciences and the College of Veterinary Medicine, University of São Paulo, Brazil (protocols # 9498230321 and # 8711260321).

Note that full information on the approval of the study protocol must also be provided in the manuscript.
