# Supplementary material for: Calpeptin is a potent cathepsin inhibitor and drug candidate for SARS-CoV-2 infections
Source: Commun Biol. 2023 Oct 18;6:1058. doi: 10.1038/s42003-023-05317-9 (PMC10584882; doi:10.1038/s42003-023-05317-9)
Supplement: Supplementary file 6 — MD checklist [file 42003_2023_5317_MOESM6_ESM.docx]

| **Reliability and reproducibility checklist for molecular dynamics simulations**  ***All boxes must be marked YES by acceptance unless “Response not needed if No”.** | | **Yes** | **No** | **Response  (Please state where this information can be found in the text)** |
| --- | --- | --- | --- | --- |
| **1. Convergence of simulations and analysis** | | | | |
| 1a. Is an evaluation presented in the text to show that the property being measured has equilibrated in the simulations (*e.g.* time-course analysis)? | |  |  | There is no such analysis in our manuscript. |
| 1b. Then, is it described in the text how simulations are split into equilibration and production runs and how much data were analyzed from production runs? | |  |  | There is no such analysis in our manuscript. |
| 1c. Are there at least 3 simulations per simulation condition with statistical analysis? | |  |  | There is no such analysis in our manuscript. |
| 1d. Is evidence provided in the text that the simulation results presented are independent of initial configuration? | |  |  | There is no such analysis in our manuscript. |
| **2. Connection to experiments** | | | | |
| 2a. Are calculations provided that can connect to experiments (*e.g.* loss or gain in function from mutagenesis, binding assays, NMR chemical shifts, J-couplings, SAXS curves, interaction distances or FRET distances, structure factors, diffusion coefficients, bulk modulus and other mechanical properties, *etc*.)? | |  |  | We have only small molecule NMR for quality control in our manuscript. |
| **3. Method choice** | | | | |
| 3a. Do simulations contain membranes, membrane proteins, intrinsically disordered proteins, glycans, nucleic acids, polymers, or cryptic ligand binding? | |  |  | Response not needed if **No** |
| 3b. Is it described in the text whether the accuracy of the chosen model(s) is sufficient to address the question(s) under investigation (e.g. all-atom vs. coarse-grained models, fixed charge vs. polarizable force fields, implicit vs. explicit solvent or membrane, force field and water model, etc.)? | |  |  | There is no such analysis in our manuscript. |
| 3c. Is the timescale of the event(s) under investigation beyond the brute-force MD simulation timescale in this study that enhanced sampling methods are needed? | |  |  | There is no such analysis in our manuscript. |
|  | If **YES**, are the parameters and convergence criteria for the enhanced sampling method clearly stated? |  |  |  |
|  | If **NO,** is the evidence provided in the text? |  |  |  |
| **4. Code and reproducibility** | | | | |
| 4a. Is a table provided describing the system setup that includes simulation box dimensions, total number of atoms, total number of water molecules, salt concentration, lipid composition (number of molecules and type)? | |  |  | There is no such analysis in our manuscript. |
| 4b. Is it described in the text what simulation and analysis software and which versions are used? | |  |  | There is no such analysis in our manuscript. |
| 4c. Are other parameters for the system setup described in the text, such as protonation state, type of structural restraints if applied, nonbonded cutoff, thermostat and barostat, etc.? | |  |  | There is no such analysis in our manuscript. |
| 4d. Are initial coordinate and simulation input files and a coordinate file of the final output provided as supplementary files or in a public repository? | |  |  | There is no such analysis in our manuscript. |
| 4e. Is there custom code or custom force field parameters? | |  |  | Response not needed if **No** |
|  | If **YES**, are they provided as supplementary files or in a public repository? |  |  |  |
